# Inducible CCR2^+^ nonclassical monocytes mediate the regression of cancer metastasis

**DOI:** 10.1172/JCI179527

**Published:** 2024-10-01

**Authors:** Xianpeng Liu, Ziyou Ren, Can Tan, Félix L. Núñez-Santana, Megan E. Kelly, Yuanqing Yan, Haiying Sun, Hiam Abdala-Valencia, Wenbin Yang, Qiang Wu, Takahide Toyoda, Marija Milisav, S. Marina Casalino-Matsuda, Emilia Lecuona, Emily Jeong Cerier, Lena J. Heung, Mohamed E. Abazeed, Harris Perlman, Ruli Gao, Navdeep S. Chandel, G.R. Scott Budinger, Ankit Bharat

**Affiliations:** 1 Division of Thoracic Surgery/Canning Thoracic Institute, Feinberg School of Medicine, Northwestern University/Northwestern Medicine, Chicago, Illinois, USA.; 2 Department of Dermatology,; 3 Division of Cardiology, Department of Medicine, and; 4 Pulmonary and Critical Care Medicine, Feinberg School of Medicine, Northwestern University, Chicago, Illinois, USA.; 5 Division of Infectious Diseases, Department of Medicine, Cedars-Sinai Medical Center, Los Angeles, California, USA.; 6 Department of Radiation Oncology,; 7 Division of Rheumatology, and; 8 Department of Biochemistry, Feinberg School of Medicine, Northwestern University, Chicago, Illinois, USA.

**Keywords:** Immunology, Cancer, Lung cancer

## Abstract

A major limitation of immunotherapy is the development of resistance resulting from cancer-mediated inhibition of host lymphocytes. Cancer cells release CCL2 to recruit classical monocytes expressing its receptor CCR2 for the promotion of metastasis and resistance to immunosurveillance. In the circulation, some CCR2-expressing classical monocytes lose CCR2 and differentiate into intravascular nonclassical monocytes that have anticancer properties but are unable to access extravascular tumor sites. We found that in mice and humans, an ontogenetically distinct subset of naturally underrepresented CCR2-expressing nonclassical monocytes was expanded during inflammatory states such as organ transplant and COVID-19 infection. These cells could be induced during health by treatment of classical monocytes with small-molecule activators of NOD2. The presence of CCR2 enabled these inducible nonclassical monocytes to infiltrate both intra- and extravascular metastatic sites of melanoma, lung, breast, and colon cancer in murine models, and they reversed the increased susceptibility of *Nod2^–/–^* mutant mice to cancer metastasis. Within the tumor colonies, CCR2^+^ nonclassical monocytes secreted CCL6 to recruit NK cells that mediated tumor regression, independent of T and B lymphocytes. Hence, pharmacological induction of CCR2^+^ nonclassical monocytes might be useful for immunotherapy-resistant cancers.

## Abstract 2

Pharmacologically induced CCR2-expressing non-classical monocytes can infiltrate and regress immunotherapy-resistant cancers by recruiting natural killer cells.

## Graphical abstract

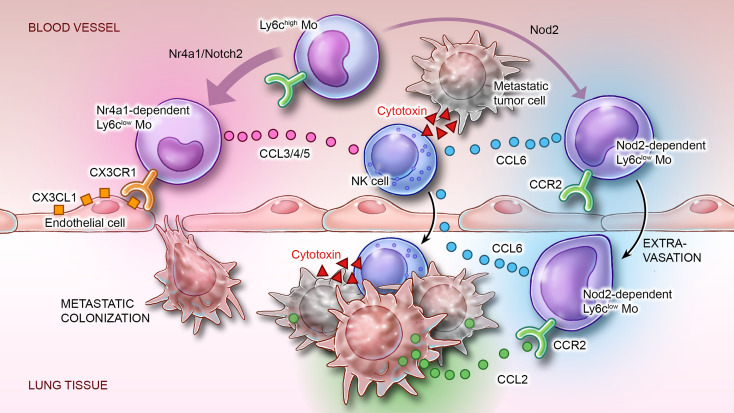

## Introduction

Tumors augment monopoiesis and orchestrate the epigenetic reprogramming of CCR2^+^ classical monocytes (CMs) into tumor-associated macrophages that drive immune suppression to promote tumor progression, metastasis, and resistance to immunotherapy (1–3). Monocytes are recruited to the tumor microenvironment via the CCR2/CCL2 axis, which has emerged as a potential target in approaches to slow cancer progression (4, 5).

Some CMs, designated CD14^++^CD16^–^ in humans and LY6C^hi^ in mice, progressively differentiate into intermediate monocytes (IntMs; CD14^++^CD16^+^ in humans and LY6C^int^ in mice) and nonclassical monocytes (NCMs; CD14^+^CD16^++^ in humans and LY6C^lo^ in mice) (6, 7). Under steady-state conditions, this process is governed by the transcription factors C/EBPβ and NR4A1, as evidenced by the monocytopenia of NCMs in *Cebpb^–/–^* or *Nr4a1^–/–^* mice (8–11). During homeostasis, these naturally occurring NCMs (N-NCMs), identified as CX3CR1^hi^CCR2^–^MHC II^lo^ by use of flow cytometry, patrol the vasculature, engaging with circulating tumor cells to mitigate metastasis (11). Nevertheless, their migration to extravascular tumors is limited due to the absence of CCR2 (8).

LY6C^int^ IntM cells, the immediate precursors of NCMs, are heterogeneous. Other studies have shown that cells within this subset demonstrate elevated CCR2, MHC II, and CD209 expression, distinguishing them both ontogenetically and phylogenetically from another cluster that is CCR2^–^MHC II^–^ and gives rise to NR4A1-dependent LY6C^lo^ N-NCMs (9). This heterogeneity, coupled with observations that the small proportion of remaining LY6C^lo^ NCMs in *Nr4a1^–/–^* mice (8) have increased CCR2 and MHC II expression, suggests an alternate NR4A1-independent pathway for the development of LY6C^lo^ NCMs. Muramyl dipeptide (MDP), an agonist of the pattern recognition receptor nucleotide-binding oligomerization domain–containing 2 (NOD2), increases the abundance of LY6C^lo^ NCMs in *Nr4a1^–/–^* mice (12); and infection from SARS-CoV-2 virus, which has single-stranded RNA (ssRNA), a natural NOD2 agonist, is associated with the development of a unique NCM subtype with C1q expression in humans. Accordingly, we hypothesized that NR4A1 and NOD2 are necessary for mutually exclusive and transcriptionally dichotomous pathways for NCM differentiation. Our data indicate that during homeostasis, NR4A1 is dominant, driving the differentiation of N-NCMs with canonical CX3CR1^hi^CCR2^–^MHC II^lo^ markers. However, NOD2 activation promoted differentiation of CMs toward an inducible NCM (I-NCM) phenotype with noncanonical CX3CR1^lo^CCR2^+^MHC II^hi^ markers. We found that these populations of NCMs performed distinct functions. NOD2-induced I-NCMs inhibited tumor seeding and induced regression of various tumor colonies more robustly than did N-NCMs. We show that unlike N-NCMs, I-NCMs migrated into both vascular and extravascular tumor microenvironments via the CCR2/CCL2 axis, where they released CCL6 to recruit NK cells that promote tumor lysis, independent of T and B lymphocytes. Collectively, our findings suggest that the induction of I-NCMs via NOD2 activation can attenuate tumor metastasis independent of conventional immune pathways necessary for immunotherapies.

## Results

### Inducible Nod2-dependent NCMs are ontogenetically distinct but are underrepresented in healthy individuals

#### Ontogenetic dichotomy of inducible NCMs and N-NCMs.

**Figure 1:**
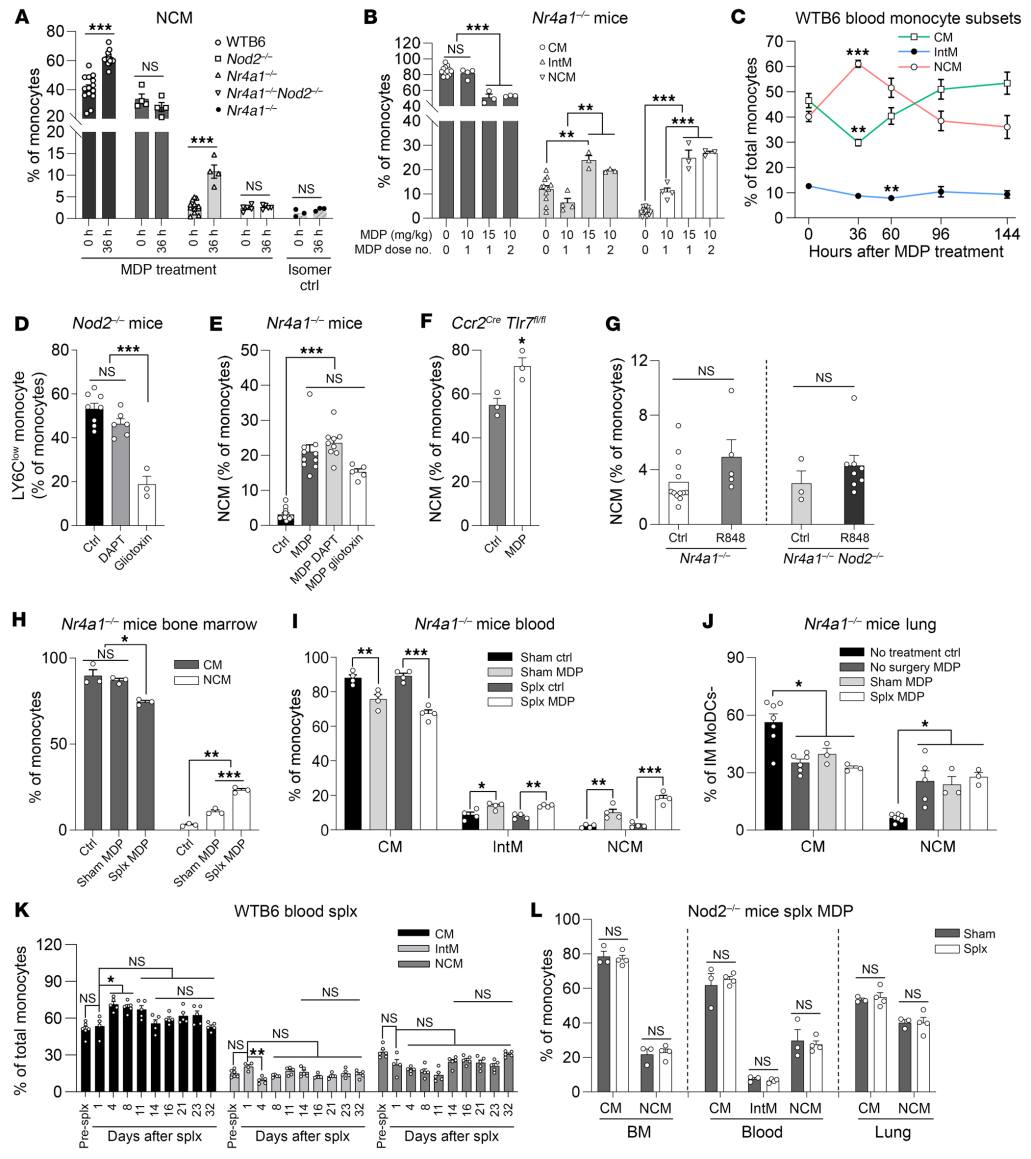
Ontogenetic dichotomy of I-NCMs and N-NCMs. (**A**–**C**) NOD2 activation in vivo by MDP but not isomer control induced conversion of blood CMs in WT and *Nr4a1^–/–^*, but not *Nod2^–/–^* or double-mutant *Nr4a1^–/–^* *Nod2^–/–^*, mice to I-NCMs (**A**), in a dose- (**B**) and time-dependent (**C**) manner. (**D** and **E**) Inhibition of the NOTCH2 signaling pathway by either gliotoxin (10 mg/kg) or DAPT (25 mg/kg) did not impair the development of I-NCMs in *Nr4a1^–/–^* mice but reduced N-NCMs in *Nod2^–/–^* mice. (**F** and **G**) Development of I-NCMs was not altered by conditional genetic depletion of TLR7 in CCR2^+^ CM or TLR7/8 activation by R848 treatment (50 μg/mouse). (**H**–**L**) Four weeks after splenectomy or sham surgery, mice were treated with MDP or control for 36 hours, and the monocyte subsets were analyzed by flow cytometry. (**H**–**J**) The increase in I-NCMs in the bone marrow (**H**), blood (**I**), and lung (**J**) was not impaired by splenectomy (Splx) in *Nr4a1^–/–^* mice. Interstitial macrophages (IMs); monocyte-derived dendritic cells (MoDCs). (**K**) The ratio of LY6C^lo^ NCMs in blood monocytes was stabilized 2 weeks after splenectomy in the blood of WT B6 mice. *n* = 5. (**L**) Ratio of monocyte subsets in total monocytes in BM, blood, and lung in *Nod2^–/–^* mice (*n* = 3–4). Unless otherwise mentioned, MDP treatment indicates 1–2 doses of 10 mg/kg MDP via retro-orbital injection for 36 hours; *n* = 3–12 mice in each group; data are presented as mean ± SEM. *P < 0.05; **P < 0.01, ***P < 0.001; 2-tailed *t* test was used for **A**, **F**, and **G**; Kruskal-Wallis (nonparametric) test for **C**: IntM and NCM, **J**: NCM, and **K**: IntM; 1-way ANOVA for the rest of the panels.

We isolated circulating LY6C^lo^ NCMs from *Nr4a1^–/–^* mice (Supplemental Figure 1A; supplemental material available online with this article; https://doi.org/10.1172/JCI179527DS1) and found that they had a larger percentage of NCMs expressing MHC II (I-A/I-E), CD14, and CCR2 (Supplemental Figure 1B), and a lower percentage of NCMs expressing CX3CR1 (Supplemental Figure 1C) and PD-L1 (CD274) (Supplemental Figure 1D), when compared with NCMs from WT mice. Induction of sterile inflammation by LPS treatment (Supplemental Figure 1, F–K) and lung or spleen transplantation (Supplemental Figure 2, A–G) resulted in a spontaneous increase in (recipient-origin) LY6C^lo^ NCMs in *Nr4a1^–/–^* mice (Supplemental Figure 1, F, G, I, and J, and Supplemental Figure 2, A–E), enabling phenotypic analysis by flow cytometry (Supplemental Figure 1, H and K) and transcriptional analysis by RNA sequencing (RNA-Seq) (Supplemental Figure 2, F and G); this confirmed high expression of *Ccr2* and MHC II genes, reminiscent of the NCM population previously reported in resting *Nr4a1^–/–^* mice (8) and those induced by the NOD2 agonist MDP (12). We hypothesized that these LY6C^lo^CX3CR1^lo^CCR2^hi^MHC II^hi^ NCMs were NOD2-dependent (referred to as inducible NCMs [I-NCMs]) and distinct from the naturally abundant NR4A1-dependent LY6C^lo^CX3CR1^hi^CCR2^lo^MHC II^–^ NCMs (N-NCMs). Treatment with MDP, but not PBS or MDP L-L isomer control (Figure 1A), induced conversion of LY6C^hi^ CMs into I-NCMs in a dose- (Figure 1B) and time-dependent (Figure 1C) manner in *Nr4a1^–/–^* and WT but not in *Nod2^–/–^* or *Nod2^–/–^ Nr4a1^–/–^* mice (Figure 1A). In contrast, while *Nr4a1^–/–^* mice lacked N-NCMs, these were abundant in *Nod2^–/–^* mice (Supplemental Figure 1, A and E). Both DAPT, a γ-secretase inhibitor, and gliotoxin, a potent NOTCH2 transactivation inhibitor, reduced N-NCM levels in *Nod2^–/–^* mice (Figure 1D) but did not prevent MDP-induced development of I-NCMs in *Nr4a1^–/–^* mice (Figure 1E). Cell-intrinsic synergistic NOTCH2 and TLR7 activation can promote the development of LY6C^lo^ NCMs from LY6C^hi^ CMs under inflammatory conditions (13). However, we found that MDP treatment was equally effective in converting LY6C^hi^ CMs into I-NCMs in *Ccr2^cre^ Tlr7^fl/fl^* mice (Figure 1F). Additionally, R848, a TLR7/8 agonist, did not promote the conversion of CMs into I-NCMs in *Nr4a1^–/–^* and *Nod2^–/–^ Nr4a1^–/–^* mice (Figure 1G). The spleen serves as a monocyte reservoir (14, 15), and DLL1, a NOTCH2 ligand and a known promoter of N-NCM development (16), is highly expressed in the marginal zone of the spleen. Hence, we tested whether the presence of the spleen was needed for the development of I-NCMs and N-NCMs. We found that MDP treatment of splenectomized *Nr4a1^–/–^* mice (Figure 1, H–J) did not alter induction of I-NCMs in the bone marrow, blood, and lungs. Similarly, in WT mice, splenectomy did not affect N-NCMs for up to 32 days (Figure 1K). Additionally, splenectomy combined with MDP treatment did not affect N-NCMs in *Nod2^–/–^* mice (Figure 1L). Hence, we conclude that the spleen is dispensable for the spontaneous development of N-NCMs as well as pharmacological induction of I-NCMs.

#### I-NCMs and N-NCMs are transcriptionally and phenotypically distinct.

**Figure 2:**
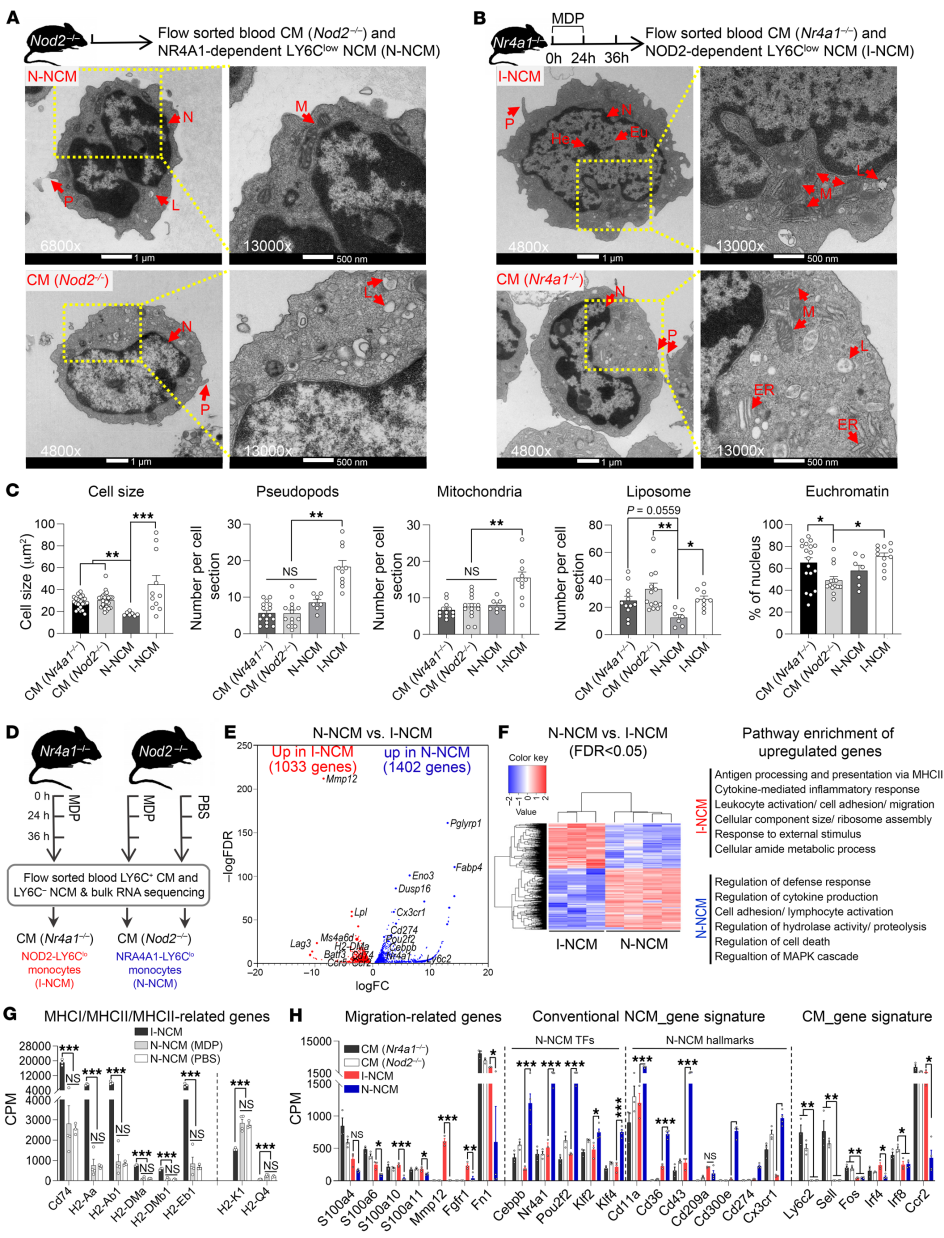
I-NCMs and N-NCMs are phenotypically and transcriptionally distinct. (**A**–**C**) Ultrastructural examination of monocyte subsets by TEM. (**A** and **B**) Experimental design and representative TEM images. Blood LY6C^hi^ or LY6C^lo^ monocyte subsets were sorted from untreated *Nod2^–/–^* or MDP-treated *Nr4a1^–/–^* mice and embedded with agarose for TEM. Scale bars, left columns: 1 μm; right column: 500 μm; magnification, left columns ×6,800; right columns, ×13,000. (**C**) Statistical analysis of cell size, number of pseudopods (P), mitochondria (M), and liposomes (L) and ratio of euchromatin (Eu) in the 4 monocyte subsets shown in **A** and **B** based on the TEM data. Approximately 7–28 cells were counted or measured in each group; data are presented as mean ± SEM; *P < 0.05, **P < 0.01, ***P < 0.001; Kruskal-Wallis test or Brown-Forsythe and Welch’s ANOVA. (**D**–**H**) Transcriptomic profiling of I-NCMs and N-NCMs. Blood samples were taken from the facial vein of MDP-treated *Nr4a1^–/–^* (I-NCMs) or *Nod2^–/–^* (N-NCMs) mice and used for RNA extraction and subsequent bulk RNA-Seq. (**D**) Experimental design schematic showing sample preparation. (**E**) Volcano plot demonstrating significantly (FDR <0.05) differentially expressed genes (DEGs) in I-NCMs and N-NCMs. (**F**) Heatmap analysis (left) demonstrated differential gene expression and pathway enrichment analysis (right) in I-NCMs and N-NCMs. (**G** and **H**) Pairwise comparison of gene expression of the selected gene signatures in blood monocyte subsets from MDP-treated *Nr4a1^–/–^* or *Nod2^–/–^* mice. TFs, transcription factors. In **G** and **H**, RNA-Seq CPM data are presented as mean ± SEM; *n* = 3-4 in each group; *P < 0.05, **P < 0.01, ***P < 0.001; 1-way ANOVA.

To obtain pure cell populations of N-NCMs and I-NCMs, we sorted N-NCMs from *Nod2^–/–^* mice and I-NCMs from MDP-treated *Nr4a1^–/–^* mice and compared their ultrastructure using transmission electron microscopy (TEM). Qualitatively, the N-NCMs were smaller than the I-NCMs (Figure 2, A–C). Furthermore, I-NCMs had abundant pseudopods, mitochondria, liposomes, and nuclear euchromatin (Figure 2, B and C) relative to N-NCMs and LY6C^hi^ CMs from *Nr4a1^–/–^* mice (Figure 2C). We compared the transcriptomes of I-NCMs and N-NCMs using bulk RNA-Seq of flow-sorted cells (Figure 2D). Pathway enrichment analysis of differentially expressed genes (Figure 2E) revealed “Leukocyte activation/cell adhesion/migration,” “Cellular component size,” and “Cellular amide metabolic process” (Figure 2F) in the I-NCMs, consistent with the analysis of cellular ultrastructure (Figure 2, A–C). Genes with increased expression in I-NCMs relative to N-NCMs included MHC II– or MHC II–related genes (*Cd74*, *H2-Aa*, *H2-Ab1*, *H2-DMa*, *H2-DMb1*, and *H2-Eb1*) (Figure 2G) and migration-related genes (*S100a4*/*6*/*10*/*11*, *Mmp12*, *Fgfr1*, and *Fn1*) (Figure 2H, left). Genes with increased expression in N-NCMs relative to I-NCMs included monocyte conversion–related transcription factors (*Cebpb*, *Nr4a1*, *pou2f2*, *Klf4*) and hallmark NCM genes (*Cd11a*, *Cd36*, *Cd43*, *Cd274*, *Cx3cr1*). Hallmark genes associated with CMs were reduced to a greater extent in N-NCMs than in I-NCMs (*Irf4*, *Ccr2*) (Figure 2H, right). In contrast, we found lower expression of *Cebpb* and *Cx3cr1* in I-NCMs compared with N-NCMs, and expression of the *Nr4a1*, *pou2f2*, *Klf4*, *Cd43*, *Cd274*, and *Ccr2* genes in I-NCMs was comparable to that in LY6C^hi^ CMs (Figure 2H, middle and right). Upregulation of transcription factors (such as *Irf4*, *Cebpa*, *Myc*, *Batf3*, *Id2*, and *Id3*) (Figure 2H and Supplemental Figure 3A, left) and differentiation-related genes (Supplemental Figure 3A, right) in I-NCMs compared with N-NCMs suggested distinct transcriptional pathways for their differentiation. Moreover, pairwise comparison revealed the expression of dendritic cell–like (Supplemental Figure 3B) or macrophage-like gene signatures (Supplemental Figure 3C) in I-NCMs relative to N-NCMs, with higher expression of the *Cd11c*, *Ifi30*, *Ly86*, *Entpd1*, and *C1q* genes. Furthermore, we found low expression of *Tlr* genes (Supplemental Figure 3D) and high expression of *Nod2* in I-NCMs (Supplemental Figure 3E). The most substantial transcriptional markers for I-NCMs in mouse blood (Supplemental Figure 3F), by which typical I-NCMscan be defined or characterized, were verified at the mRNA level using quantitative RT-PCR (qPCR) (Supplemental Figure 3G) and at the protein level using flow cytometry (Supplemental Figure 1, B–D). The transcriptional changes in LY6C^lo^ I-NCMs isolated from MDP-treated *Nr4a1^–/–^* mice were not artificially induced by pharmacological manipulation, as administration of MDP did not alter the transcriptional profile of LY6C^lo^ NCMs in *Nod2^–/–^* mice (Supplemental Figure 3, H and I). Additionally, as expected, I-NCMs were distinct from both LY6C^int^ and LY6C^hi^ monocytes from the same host (Supplemental Figure 4). Total LY6C^lo^ NCMs in naive WT and *Nod2^–/–^* mice were transcriptionally indistinguishable (Supplemental Figure 3I) and resembled the pure N-NCM population, suggesting that the low abundance of these cells precludes the deconvolution of their data from the bulk RNA-Seq data.

When we treated *Nr4a1^–/–^* mice with MDP, we observed expansion of LY6C^lo^ NCMs, with a homogeneous population expressing similar markers to those of I-NCMs. In contrast, *Nod2^–/–^* or *Nod2^–/–^ Nr4a1^–/–^* mice did not exhibit any expansion of LY6C^lo^ NCMs after MDP treatment (Figure 1A and Supplemental Figure 1E). The natural abundance of N-NCMs is further illustrated by our inability to characterize typical I-NCM markers using transcriptional profiling of blood LY6C^lo^ NCMs isolated from MDP-treated WT mice (Supplemental Figure 5, A and B) and the stark differences between the pure I-NCM population and total WT MDP–treated blood LY6C^lo^ NCMs (Supplemental Figure 5A and Supplemental Figure 5, C and D). Taken together, these data suggest that N-NCMs and I-NCMs represent distinct subsets of NCMs, and while the latter are naturally underrepresented, they can be readily induced in response to NOD2 signaling.

#### Reconciliation with reported phenotypes of NCMs.

NOD2 signaling can be triggered by ssRNA viruses such as coronaviruses (17). The high expression of *C1q* genes in I-NCMs in mouse blood (Supplemental Figure 3C) was reminiscent of the enrichment of *C1q* genes reported in C1 monocytes identified in the blood of COVID-19 patients (18). We compared the expression of typical markers of monocytes in C1 monocytes and conventional NCMs in human blood monocyte scRNAseq data. CCR2 and CX3CR1 are typical markers for mouse monocytes, while CD14, HLA-DR and CD16 are typical markers for human monocytes. CCR2 is highly expressed in mouse CM and I-NCM, while its expression in general is relatively low in human blood monocytes. In human C1-NCM in human blood, the expression of CCR2 is higher compared to that in conventional NCM (CD16^+^ NCM) in Supplemental Figure 6A. Hence, we hypothesized that C1 monocytes are human homologs of murine NOD2-dependent LY6C^lo^ NCMs. To test this hypothesis, we performed a similarity analysis between mouse blood NOD2-dependent LY6C^lo^ I-NCMs and human blood C1 monocytes using a short list of 20 marker genes that are upregulated and commonly expressed in both species (Supplemental Table 1), as well as a long list (2,408 genes) of all upregulated genes in NOD2-dependent LY6C^lo^ I-NCMs (>1.5-fold compared with NR4A1-dependent LY6C^lo^ N-NCMs) (Supplemental Table 2). The similarity analysis demonstrated that genes characterizing NOD2-dependent LY6C^lo^ I-NCMs were enriched in C1 monocytes reported in blood samples from COVID-19–affected humans (Supplemental Figure 6, B and C), including MHC II genes such as HLA-DR (Supplemental Figure 6A). To further determine whether ssRNA in SARS-CoV-2 can promote the development of I-NCMs in mice, we isolated RNA from SARS-CoV-2 and murine influenza A virus and obtained commercially available synthetic ssRNA. We found that synthetic ssRNA and COVID-19 ssRNA, but not influenza ssRNA, increased the population of I-NCMs (Supplemental Figure 6, D–G). To investigate the clinical relevance of our findings — i.e., whether NOD2 activation could induce development of a homologous population of I-NCMs in humans — we cultured freshly isolated CD14^+^ monocytes with MDP or liposomal muramyl tripeptide phosphatidyl ethanolamine (L-MTP-PE, mifamurtide) (Supplemental Figure 7, A–D). Within 24 hours, we observed conversion of CD14^++^CD16^–^HLA-DR^dim/–^ CMs into CD14^+^CD16^dim/–^HLA-DR^++/+^ I-NCMs (Supplemental Figure 7, E and F).

NCMs with high expression of PD-L1 have been reported in circulating blood from normal mice (19). To determine whether the PD-L1–expressing NCMs were NR4A1- or NOD2-dependent, we isolated PD-L1^neg^ and PD-L1^pos^ LY6C^lo^ NCMs in the resting state from WT mice and performed RNA-Seq (Supplemental Figure 8A). While the subsets were transcriptionally distinct (Supplemental Figure 8, B and C), PD-L1^neg^ NCMs showed characteristics of I-NCMs (Supplemental Figure 8, D–G), while PD-L1^pos^ NCMs resembled N-NCMs. Furthermore, steady-state PD-L1^neg^LY6C^lo^ NCMs and MDP-induced NOD2-dependent LY6C^lo^ I-NCMs isolated from *Nr4a1^–/–^* mice were transcriptionally similar (Supplemental Figure 8, B–H).

### Increased susceptibility to cancer metastasis in *Nod2^–/–^* mice is reversed by I-NCMs

**Figure 3:**
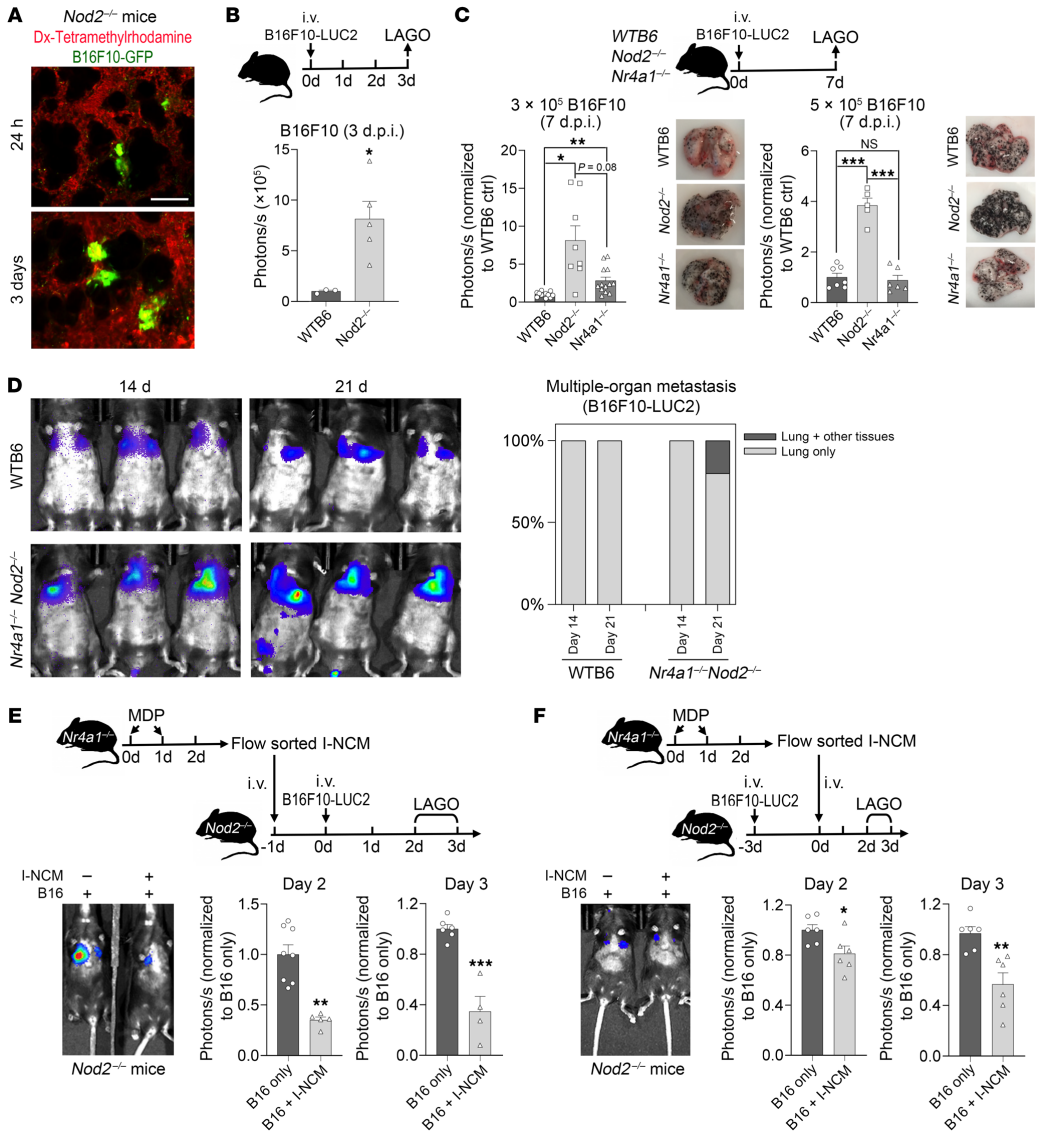
I-NCMs mediate the regression of melanoma metastasis. (**A**) Representative 2-photon images showing accumulation and colonization of B16F10-GFP in the lung at the indicated time points after retro-orbital injection into *Nod2^–/–^* mice. Scale bar: 100 μm. Dx, dextran (**B**) Accumulation of B16F10-LUC2 cells was detected and determined by luciferase activity using LAGO on day 3 after retro-orbital injection (d.p.i.). (**C**) Detection of melanoma cells in lungs in WT B6, *Nr4a1^–/–^*, and *Nod2^–/–^* mice by LAGO at the indicated dose and time points. The lung was isolated and photographed to visualize the accumulation of black dots in the metastatic B16F10-LUC2 cells after LAGO detection. (**D**) Metastatic B16F10 colonization in lung and other tissues was analyzed in monocyte-depleted mice. (**E** and **F**) Adoptive transfer of I-NCMs reduced metastatic colonization (**E**) and attenuated established metastatic B16F10 melanoma colonies (**F**). Approximately 1 × 10^5^ I-NCMs from MDP-treated *Nr4a1^–/–^* mice were retro-orbitally injected into *Nod2^–/–^* mice prior to or after the injection of 3 × 10^5^ B16F10-LUC2 cells. Colonization of B16 in the lung was determined by LAGO detection of luciferase activity. In **E** and **F**, the experimental design is shown in the upper panel; a representative image and quantification of LAGO detection of luciferase activity in B16F10-LUC2 cells in the lung are shown in the lower-left panel and lower-right panel, respectively. The data are presented as mean ± SEM; *n* = 3–13 in each group; *P < 0.05, **P < 0.01, ***P < 0.001; 2-tailed t test in **B**, **E**, and **F**; 1-way ANOVA in **C**.

**Figure 4:**
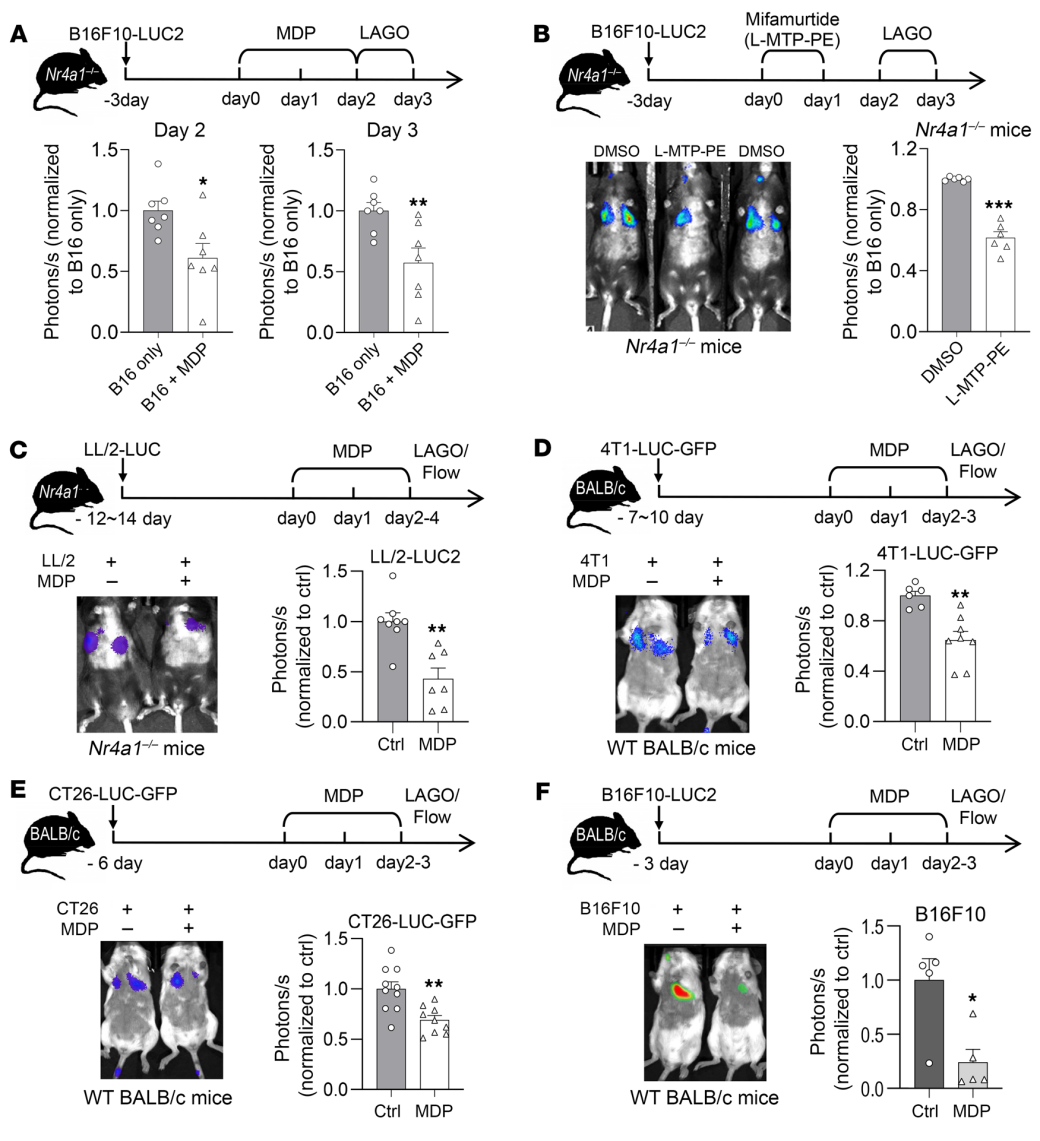
Pharmacological induction of I-NCMs promotes regression of hematogenous metastasis from multiple cancers. MDP or L-MTP-PE attenuates metastatic colonization of multiple cancers. (**A** and **B**) MDP (**A**) or the liposome-conjugated MDP analog MTP (L-MTP-PE, or mifamurtide) (**B**) reduced the number of established B16F10 clusters in *Nr4a1^–/–^* mice. (**C**) MDP treatment attenuated the accumulation of established LL/2-LUC lung cancer cells in *Nr4a1^–/–^* mice. (**D**–**F**) MDP treatment attenuated accumulation of the established breast cancer 4T1-LUC-GFP (**D**) or colon cancer CT26-LUC-GFP (**E**), or melanoma B16F10-LUC2 (**F**) in WT BALB/c mouse lung. Data are presented as mean ± SEM; *n* = 5–10 mice in each group; *P < 0.05, **P < 0.01, ***P < 0.001; 2-tailed *t* test in **A**–**F**.

*Nod2* deficiency has been shown to promote tumorigenesis due to the failure of intrinsic tissue regulation of inflammation (20). However, the role of *Nod2* deficiency in cancer metastasis remains unknown. In a widely used model of hematogenous metastasis in which syngeneic B16F10 melanoma cells are injected i.v. or retro-orbitally (21), deficiency of *Nod2* dramatically increased tumor metastasis to the lung and other sites, such as the liver (Supplemental Figure 9). Multiphoton intravital imaging of B16F10-GFP cells (Figure 3A) and LAGO in vivo imaging of B16F10-LUC2 cells (Figure 3B) demonstrated that melanoma cells formed clusters in the lungs within 24 hours of i.v. injection. *Nod2^–/–^* and *Nr4a1^–/–^* *Nod2^–/–^* double-mutant mice showed a pronounced increase in tumor seeding and progression of melanoma metastasis in the lungs compared with *Nr4a1^–/–^* and WT mice (Figure 3, C and D). Reconstitution of *Nod2^–/–^* mice with a pure cell population of I-NCMs prior to injection of B16F10-LUC2 melanoma cells suppressed new pulmonary B16 melanoma metastasis (Figure 3E). Furthermore, reconstitution with I-NCMs also significantly reduced the size of established pulmonary B16 melanoma metastatic colonies (Figure 3F). In contrast, reconstitution with an equal number of LY6C^lo^ NCMs from MDP-treated WT mice that contained a mixture of both N-NCMs and I-NCMs (Supplemental Figure 10A vs. Figure 3E) or pure N-NCMs from *Nod2^–/–^* mice (Supplemental Figure 10B vs. Figure 3F) was less effective in mediating the regression of pulmonary metastasis. Similarly, MDP (Figure 4, A and F) or L-MTP-PE (Figure 4B) induced regression of established pulmonary cancer colonies through the development of I-NCMs (Figure 1, A, B, and J, and Supplemental Figure 11). MDP treatment was ineffective in *Nod2^–/–^* and *Ccr2^–/–^* mice with established pulmonary metastatic melanoma colonies (Supplemental Figure 10, B and C), demonstrating the necessity of I-NCMs in MDP-mediated tumor regression. Importantly, we found that I-NCMs were broadly effective in reducing cancer metastasis, as MDP treatment induced regression of established colonies of lung, breast, and colon cancers in WT and *Nr4a1^–/–^* hosts (Figure 4, C–E). However, MDP treatment was ineffective in promoting regression of nonvascularized subcutaneously injected B16F10 tumors (Supplemental Figure 12).

### Nod2-dependent Ly6c^lo^ monocytes infiltrate tumor colonies via the CCR2/CCL2 axis and recruit NK cells through CCL6 release to mediate tumor regression

**Figure 5:**
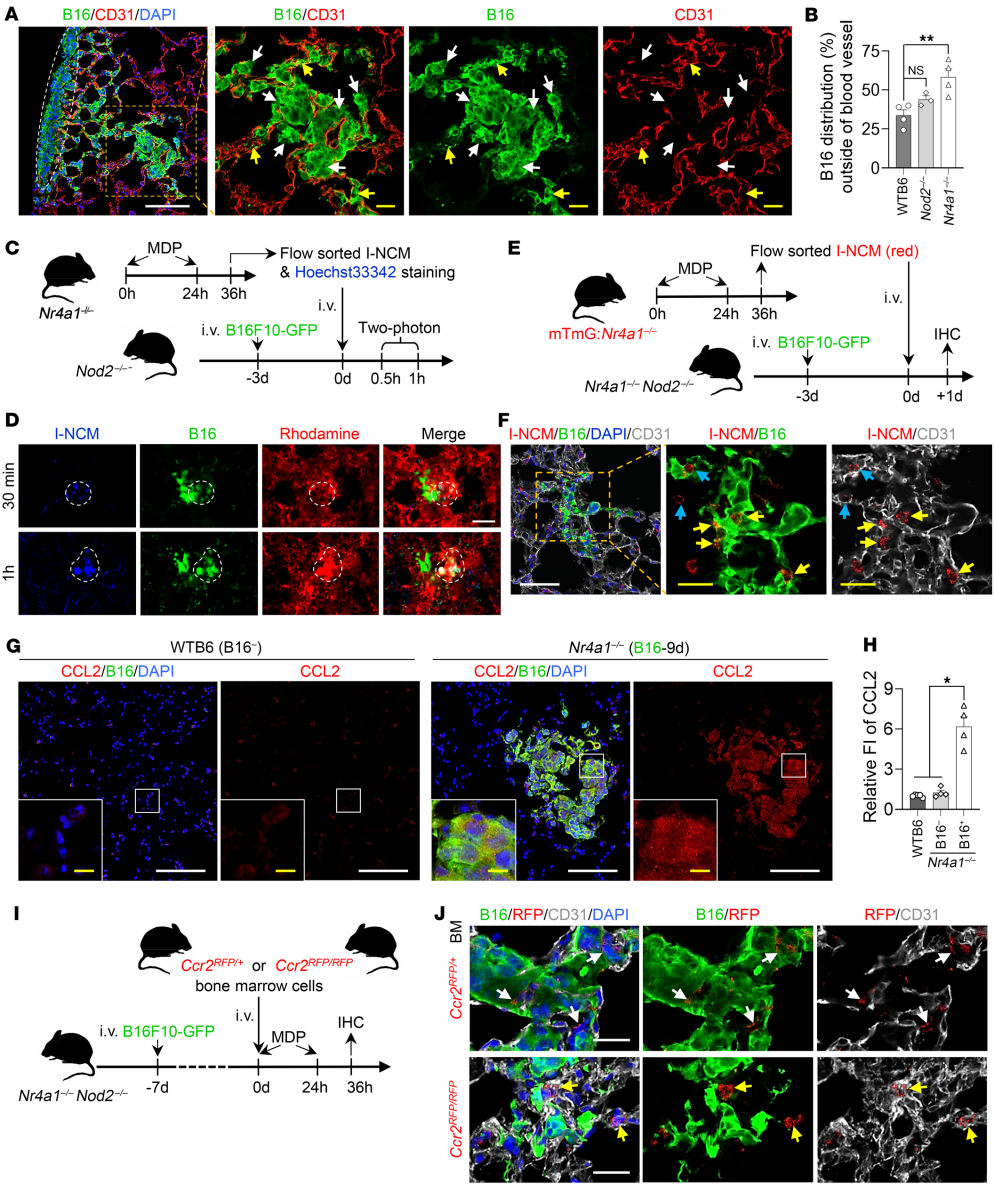
Retention of CCR2 on I-NCMs is crucial for their migration to tumor metastasis. (**A** and **B**) Intravascular and extravascular distribution of established B16F10-GFP clusters in mouse lung. (**A**) Representative confocal images show immunostaining of CD31 (endothelial cell marker, red) and B16-GFP (green) in *Nr4a1^–/–^* mice. B16F10-GFP clusters on the lung surface are indicated by dashed white lines. White and yellow arrows indicate the areas of extravascular and intravascular B16F10-GFP cells, respectively. White and yellow scale bars: 100 and 20 μm, respectively. (**B**) GFP intensity in 10–22 areas from multiple slides from the deeper regions in lung from WT B6, *Nod2^–/–^*, and *Nr4a1^–/–^* mice (*n* = 3–4) was determined by use of ImageJ and quantified. Data are presented as mean ± SEM; **P < 0.01; 1-way ANOVA. (**C** and **D**) Experimental design (**C**) and representative 2-photon images (**D**) showing colocalization of I-NCMs with the established B16F10-GFP metastatic clusters in mouse lung. The I-NCMs were sorted, stained with Hoechst 33342, and adoptively transferred into *Nod2^–/–^* mice bearing B10F10-GFP cells in the lung as described in **C**. Intravital 2-photon imaging was used for tracing I-NCMs. Blue: Hoechst 33342–stained I-NCMs; green: B16F10-GFP; red: Dx-rhodamine–labeled blood vessel. Colocalization of blue and green signals is indicated in the area circled with a dashed white line in **D**. Scale bars: 50 μm. (**E** and **F**) RFP-labeled I-NCMs (3.5 × 10^5^ cells) were sorted and adoptively transferred into *Nr4a1^–/–^ Nod2^–/–^* double-mutant mice bearing B10F10-GFP cells in the lung as described for **E**. Accumulation and distribution of the adoptively transferred RFP–I-NCMs were detected and determined by confocal imaging of lung sections immunostained with CD31 (endothelial cell marker; white), GFP (B16F10-GFP), and RFP (RFP-I-NCMs) in **F**. Yellow and blue arrows indicate the typical RFP-labeled I-NCMs outside and inside the blood vessel within the B16F10-GFP clusters, respectively. White and yellow scale bars: 50 and 20 μm, respectively. (**G** and **H**) Expression of CCL2 in metastatic B16F10 clusters and adjacent areas in mouse lungs. Expression of CCL2 was detected by IHC in the lungs (**G**) of untreated WT B6 control mice (*n* = 5, left) and *Nr4a1^–/–^* mice bearing B16F10-GFP melanoma cells (*n* = 4, right). White and yellow scale bars: 100 and 10 μm, respectively. RFP intensity in 34–96 areas from multiple slides was measured by use of ImageJ and quantified (**H**). FI, fluorescence intensity. The FI data are presented as mean ± SEM; *n* = 4–5 mice in each group; *P < 0.05; Forsythe’s and Welch’s ANOVA. (**I** and **J**) CCR2 deficiency impaired the accumulation and extravasation of MDP-triggering CCR2^+^ monocytes from blood vessels at the B16F10 metastasis site. Experimental design (**I**) and representative confocal images (**J**) showing typical extravasated (**J**, top row, indicated with white arrows) or intravascular (**J**, bottom row, indicated with yellow arrows) MDP-triggering CCR2^+^ monocytes in *Nr4a1^–/–^Nod2^–/–^* mouse lungs. Scale bars: 20 μm.

Using high-resolution imaging, we found B16F10 clusters in both the lung periphery (Figure 3C and Figure 5A) and deeper lung parenchyma. In the lung periphery, we found that all B16F10 cells were extravascular (Figure 5A; indicated with a dashed white line). In contrast, in the deeper regions, we found 30%–60% of B16F10 melanoma cells in the extravascular space, while the rest remained intravascular. We found more extravascular B16F10 melanoma cells in *Nod2^–/–^* or *Nr4a1^–/–^* than in WT mice, indicating increased engraftment (Figure 5B). When we adoptively transferred Hoechst 33342–labeled NOD2-dependent LY6C^lo^ I-NCMs isolated from MDP-treated *Nr4a1^–/–^* mice into *Nod2^–/–^* mice (Figure 5C), the cells accumulated in B16F10 clusters in the lung within 30 minutes of injection (Figure 5D and Supplemental Video 1). To study their trafficking into the established pulmonary metastatic B16F10-GFP clusters at greater resolution, at greater resolution, we generated brightly RFP-labeled NOD2-dependent LY6C^lo^ I-NCMs from MDP-treated mT/mG:*Nr4a1^–/–^* mice, isolated the RFP-labeled I-NCMS, and infused them into *Nr4a1^–/–^ Nod2^–/–^* mice (Figure 5E). We found that the greatest concentration of RFP-expressing I-NCMs was in the extravascular space within B16F10 clusters (Figure 5F). Given the high expression of CCR2 (Figure 2H and Supplemental Figure 1B) and migration-related genes (Figure 2H) in NOD2-dependent LY6C^lo^ I-NCMs, we hypothesized that signaling through CCL2/CCR2 plays a role in I-NCM–induced tumor regression. Indeed, we found that B16F10 cells had high intrinsic expression of CCL2 that increased upon their engraftment within the lungs (Figure 5, G and H, and Supplemental Figure 13A). CT26, 4T1, and LL/2 cells showed similar patterns of CCL2 expression (Supplemental Figure 13, A–C). To test whether CCR2 deficiency impaired migration of I-NCMs into tumor colonies, we used *Ccr2^RFP/RFP^* mice, in which the knockin of RFP precluded Ccr2 transcription, or *Ccr2^RFP/+^* control mice. *Ccr2^RFP/RFP^* mice are monocytopenic, as CCR2 deficiency prevents the egress of LY6C^hi^ CMs, the precursors of LY6C^lo^ NCMs, from bone marrow, while *Ccr2^RFP/+^* mice are phenotypically normal (22, 23). To determine the specific role of CCR2 in the migration of I-NCMs into tumor colonies and overcome the inability of CMs to exit the bone marrow in *Ccr2^RFP/RFP^* mice, we reconstituted *Nr4a1^–/–^ Nod2^–/–^* mice with established pulmonary B16F10-GFP metastasis with bone marrow from *Ccr2^RFP/RFP^* or *Ccr2^RFP/+^* mice (Figure 5I and Supplemental Figure 14, A and D) and treated these mice with MDP. In *Ccr2^RFP/+^* mice, while RFP is abundantly expressed on monocytes, a small population (<2%) of NK cells and dendritic cells and a minority (<10%) of neutrophils are also RFP positive (23–27). However, the lifespan of neutrophils and LY6C^hi^ CMs is shorter (34–48 hours), with a half-life of approximately 20 hours, than that of LY6C^lo^ monocytes, which persist for 5–7 days (28–30). Therefore, after 36 hours of MDP treatment following adoptive transfer of bone marrow (Figure 5I and Supplemental Figure 14, A and D), RFP was present almost exclusively on I-NCMs.

**Figure 6:**
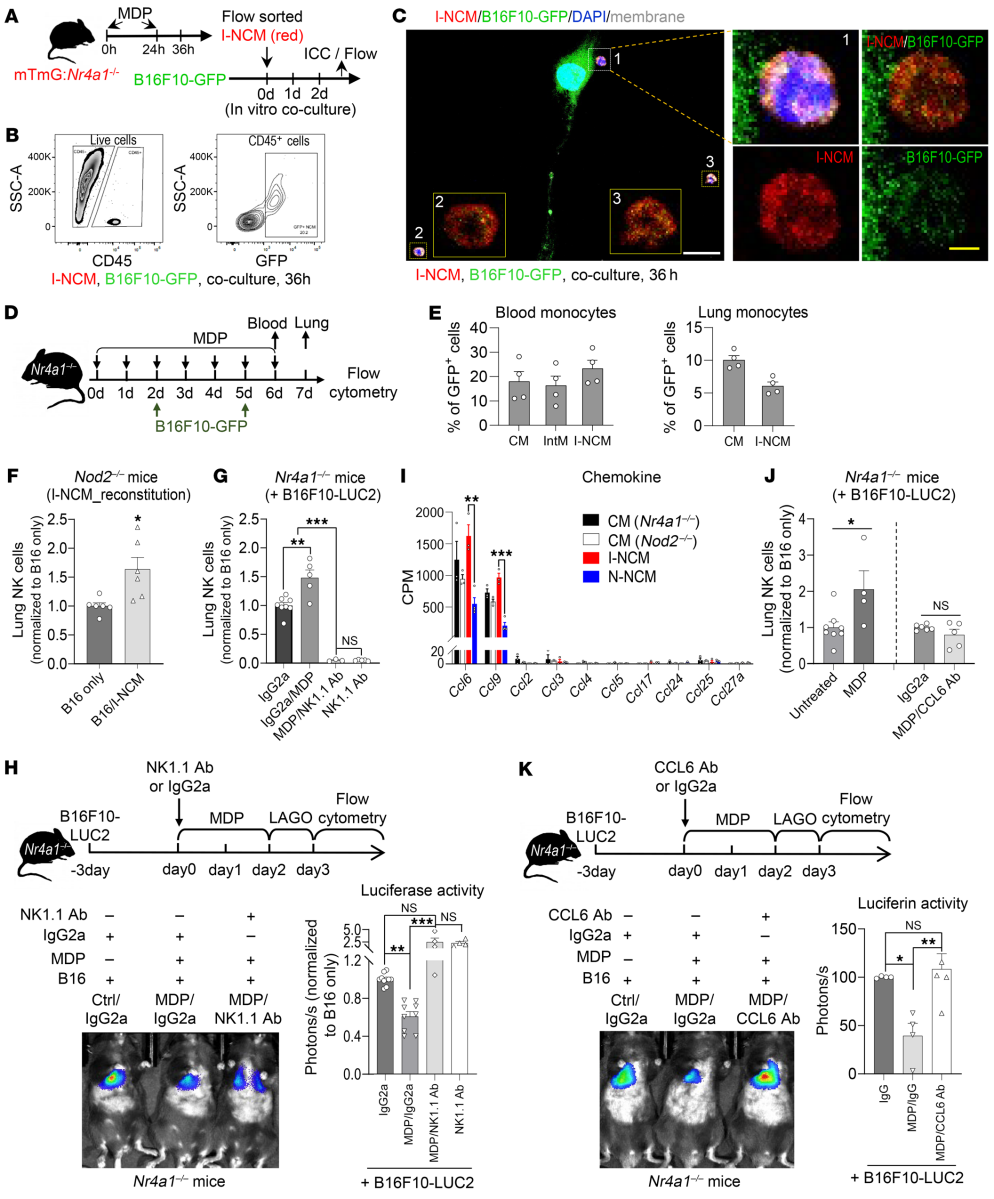
I-NCMs recruit NK cells to promote tumor regression. (**A**–**E**) Detection of the uptake of B10F10-GFP by I-NCMs in vitro (**A**–**C**) and in vivo (**D** and **E**). Experimental design (**A**) and uptake of B16F10-GFP by RFP-labeled splenic (**B**) or blood (**C**) I-NCMs at 36 hours following coculture. The GFP signal in I-NCMs was detected by flow cytometry (**B**) or ICC (**C**). Monocytes and B16 cells were cocultured at a ratio of 5:1. White and yellow scale bars: 100 and 10 μm, respectively, in **C**. Experimental design (**D**) and FACS detection of uptake of B16F10-GFP materials by monocyte subsets in blood (**E**, left) or lung (right) in *Nr4a1^–/–^* mice after retro-orbital injection of MDP and B16F10-GFP injection. (**F**–**K**) I-NCMs recruit NK cells through CCL6 release to sites of B16F10 melanoma metastasis. (**F** and **G**) Adoptive transfer of I-NCMs (**F**) and MDP treatment (**G**) increased NK cells in the lungs of B16F10-bearing *Nod2^–/–^* and *Nr4a1^–/–^* mice, respectively. (**H**) MDP-triggered attenuation of B16F10 colonization in *Nr4a1^–/–^* mice was inhibited by depletion of NK cells using NK1.1 antibody. (**I**) Bulk RNA-Seq data showing higher expression of *Ccl6* and *Ccl9*, but not other detected chemokine genes, in I-NCMs. (**J**) Anti-CCL6 antibody reduced NK cells and (**K**) suppressed the MDP-mediated attenuation of B16F10 colonization in *Nr4a1^–/–^* mice. Data are presented as mean ± SEM; *n* = 4–10 in each group; *P < 0.05, **P < 0.01, ***P < 0.001; 2-tailed *t* test in **F** and **J**, Kruskal-Wallis test in **H**, and 1-way ANOVA in **G**, **I**, and **K**.

We found that in control mice that did not receive MDP treatment, there were no RFP^+^ cells around tumor colonies (Supplemental Figure 14B). Following MDP treatment, we found RFP^+^ cells around cancer colonies in *Nr4a1^–/–^ Nod2^–/–^* mice that received either *Ccr2^RFP/RFP^* or *Ccr2^RFP/+^* bone marrow (Figure 5 and Supplemental Figure 14, C and E). However, we found that only RFP^+^ I-NCMs from *Ccr2^RFP/+^* bone marrow extravasated and infiltrated into the extravascular tumor colonies (Figure 5J, top row), while RFP^+^ cells from *Ccr2^RFP/RFP^* BM cells remained intravascular (Figure 5J, bottom row, and Supplemental Figure 14C). Since B16F10 melanoma cells can have some expression of CCR2 (31), we additionally characterized the coexpression of CCR2 and RFP around the extravascular B16F10-GFP cluster, which would only be present in I-NCMs. We found CCR2-RFP expression around melanoma cells only in mice that received BM cells from *Ccr2^RFP/+^* (Supplemental Figure 14E, top row) mice, but not *Ccr2^RFP/RFP^* (Supplemental Figure 14E, lower panel) mice, confirming that knockin mutation of *Ccr2^RFP/RFP^* prevents egress of I-NCMs into the established metastatic colonies. Unlike N-NCMs, I-NCMs did not show primary uptake of labeled tumor cell particles either in vitro (Figure 6, A–C) or in vivo (Figure 6, D and E, and Supplemental Figure 15), suggesting a limited phagocytotic function of I-NCMs in clearing tumor materials. Moreover, no appreciable direct tumor cytotoxicity by I-NCMs was observed (Supplemental Figure 16). Instead, we found a significant increase in NK cells in metastatic melanoma colonies after NOD2-dependent LY6C^lo^ I-NCMs were adoptively transferred into *Nod2^–/–^* mice (Figure 6F) or after MDP treatment of *Nr4a1^–/–^* mice (Figure 6G). Consistent with this observation, we found that the regression of MDP-triggered metastatic B16F10 colonies was reversed by the depletion of NK cells (Figure 6H).

Our comparison of I-NCMs with N-NCMs using RNA-Seq identified relatively high expression of *Ccl6* and *Ccl9* in I-NCMs (Figure 6I). While CCL9 can regulate the function of T cells (32, 33), CCL6 has been reported to be involved in the recruitment of NK cells (34). Anti-CCL6, but not anti-CCL9, antibody (Supplemental Figure 17) reduced the recruitment of NK cells (Figure 6J) and prevented tumor regression by MDP in *Nr4a1^–/–^* mice (Figure 6K), indicating that I-NCMs promote tumor regression by the CCL6-induced recruitment of NK cells. We further explored the effects of CCL6 and CCL9 on chemokine and activating/inhibitory/licensing receptors on NK cells. Although one chemokine can interact with multiple receptors, and vice versa, the receptors for CCL6 and CCL9 are not well understood. CCL6 may interact with CCR1, CCR2, and CCR3. Mouse NK cells are reported to express CCR2, CCR5, CCR7, and CCR9 (35). Our data suggest that cell-surface expression of CCR1, CCR2, CCR5, CCR7, and CCR9 on mouse lung NK cells is extremely low (Supplemental Figure 17B). No significant alteration in expression of these receptors was observed after MDP treatment or CCL9 neutralization in *Nr4a1^–/–^* mice with B16F10 lung metastasis. These data suggest that MDP treatment does not significantly alter expression of these chemokine receptors on mouse NK cells at the protein level (Supplemental Figure 17B). NK cells can express multiple activating/inhibitory/licensing receptors such as NKG2A/C/D/E, CD16, NKp46, NK1.1 (NKR-P1C), and Ly49A/C/D/H/I (36). Among them, NK1.1 and NKG2D are activating receptors, and NKG2A and Ly49C/I are related to the education or licensing of NK cells in response to stimuli such as cancer metastasis (37, 38). While MDP treatment increased levels of the activating receptor NK1.1 and/or licensing-related receptor NKG2A on blood and lung NK cells, neutralization of CCL9 did not affect upregulation of these receptors (Supplemental Figure 17, C and D). Together, these data suggest that CCL9 has no significant effect on the function of NK cells in our models. Collectively, our data suggest that expression of CCL6, but not CCL9, enhances the recruitment and activity of NK cells and contributes to I-NCM–mediated attenuation of lung metastasis.

### T lymphocytes and other immune cells are dispensable for I-NCM–dependent tumor lysis

CCL6 can recruit T cells, B cells, NK cells, and monocytes/macrophages (34, 39–42). Although MDP treatment increased the levels of I-NCMs and NK cells, it did not alter T lymphocytes in the tumor microenvironment (Supplemental Figure 17, E and F). To study other immune cells in our system, we examined the composition of immune cells in the tumor microenvironment at both early (5–7 days) (Supplemental Figure 18, B–E) and late stages (4 weeks) (Supplemental Figure 18A). We consistently found that monocytes, NK cells, eosinophils, macrophages, neutrophils, and T cells were the dominant constituents of the tumor microenvironment. Neutralization of CCL6 did not alter the numbers of neutrophils, eosinophils, alveolar macrophages, and interstitial macrophages (Supplemental Figure 18, B–E), or macrophage polarization (Supplemental Figure 19). Furthermore, no direct tumor-killing effect by eosinophils, alveolar macrophages, and interstitial macrophages was observed (Supplemental Figure 18F), although they could phagocytose tumor material from lysed cells.

**Figure 7:**
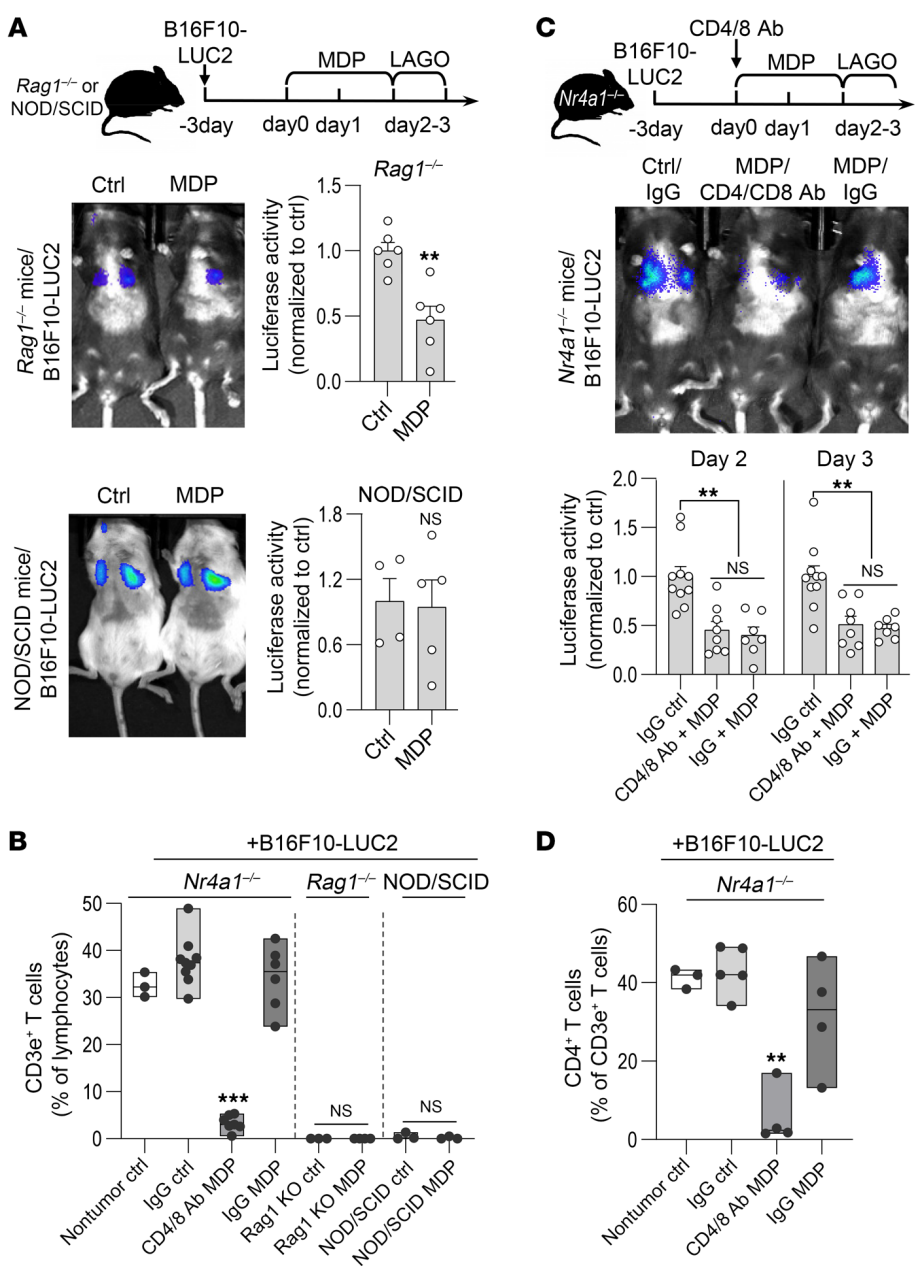
Regression of metastasis by I-NCMs is independent of T and B lymphocytes. (**A** and **B**) MDP treatment reduced the established B16F10 clusters in the lung in T cell–deficient *Rag1^–/–^* mice but not in T cell–deficient and NK-impaired NOD/SCID mice. (**C** and **D**) MDP treatment suppressed the established B16F10 clusters in *Nr4a1^–/–^* lungs (**C**) in the absence of CD4^+^ and CD8^+^ T cells (**D**). Data are presented as mean ± SEM; *n* = 3–10 in each group; **P < 0.01, ***P < 0.001; 2-tailed *t* test in **A** and **B** (for 2-column comparison), 1-way ANOVA in **B** (for multiple-column comparison) and **C** and **D**.

As we did not observe an increase in T cells at the sites of tumor lysis following MDP treatment, we next investigated whether I-NCMs can mediate regression of tumor metastasis independent of T and B lymphocytes. Accordingly, we developed tumor metastasis in *Rag1^–/–^* and NOD/SCID mice. While B and T cells are deficient in both strains, the latter also have impairment of NK cells (43–45). We found that MDP treatment reduced the metastatic colonization of B16F10 cells in *Rag1^–/–^* but not NOD/SCID mice (Figure 7, A and B), suggesting that T and B lymphocytes are dispensable for the tumor immunity mediated by I-NCMs. Next, to specifically investigate whether I-NCMs can induce tumor lysis independent of T cells, we pharmacologically depleted CD4^+^ and CD8^+^ T cells in *Nr4a1^–/–^* mice and evaluated the efficacy of I-NCMs (Figure 7, C and D). MDP treatment attenuated the metastatic colonization of B16F10 cells in T cell–deficient *Nr4a1^–/–^* mice with similar efficacy (Figure 7C).

## Discussion

In this study, we showed that LY6C^hi^ CMs gave rise to 2 transcriptionally distinct subsets of LY6C^lo^ NCMs through independent pathways of cell differentiation. These subsets exhibited phenotypic and transcriptional differences. The I-NCM subset was characterized by low CX3CR1 and high CCR2 expression. The I-NCMs were distinct from both CMs and NR4A1-dependent N-NCMs but had transcriptional similarities to previously described populations of human C1 monocytes and murine PD-L1–negative NCMs. While NR4A1-dependent N-NCMs are naturally abundant under steady-state conditions in WT hosts, the NOD2-dependent subset can be pharmacologically induced to enhance tumor immunity against a variety of metastases. Hence, we provide an ontogenetic lineage of NCMs, harmonizing the different phenotypes of LY6C^lo^ monocytes previously described in mice and humans, including CD274- and C1q-expressing and NOD2-dependent LY6C^lo^ monocytes. A variety of bioinformatics tools, scRNA-Seq, genetic knockout models, and high-resolution in vivo imaging, revealed evidence of 2 distinct lineages of NCMs: one naturally occurring and the other inducible. We reconcile the previously described monocytic populations with these 2 subsets, offering evidence of their transcriptional and phenotypic dichotomy as well as their biological relevance. Our work identifies a potentially clinically actionable pathway that could be pharmacologically manipulated to expand I-NCMs to target metastasis from various cancers.

It is well accepted that transcription factors such as C/EBPβ, NR4A1, and POU2F2 are critical for the development of NR4A1-dependent LY6C^lo^ N-NCMs from LY6C^hi^ CMs (7). Our RNA-Seq analysis suggests that alternate transcription factors, including C/EBPα, BATF3, IRF4, ID2, and ID3, may play a role in the development of NOD2-dependent LY6C^lo^ I-NCMs. NOD2 can trigger NOTCH1 signaling in macrophages (46, 47) to activate downstream inflammatory and antiinflammatory effects. Activation of NOTCH2 signaling by its ligand DLL1, on the other hand, has been shown to promote the transformation of LY6C^hi^ CMs into LY6C^lo^ NCMs by specific niches of the endothelium, including those within the arteries and marginal zone of the spleen, under steady-state conditions (16). Moreover, NOTCH2 and TLR7 signaling can promote the development of LY6C^lo^ NCMs from LY6C^hi^ CMs under inflammatory conditions (13, 16). Our findings are consistent with these prior observations but demonstrate that NOTCH2- and TLR7-dependent conversion plays a role in NR4A1-dependent but not NOD2-dependent LY6C^lo^ NCMs.

NOD2 can bind directly to the bacterial peptidoglycan MDP, which is present in both Gram-positive and Gram-negative bacteria (48, 49), triggering an immune reaction mediated through NF-κB (50, 51). NOD2 signaling may also be triggered by other stimuli, such as ssRNA viruses (17). The similarity between the I-NCMs we describe here and COVID-19–affected human blood C1q-espressing NCMs raises the possibility that they may be homologous, as SARS-CoV-2 is an ssRNA virus. Our data demonstrating that ssRNA from SARS-CoV-2 virus, but not influenza A, can induce murine I-NCMs and that treatment of human CMs with NOD2 agonists converts them into I-NCMs are supportive of this hypothesis. Our findings are consistent with previous observations that CCR2^hi^MHC II^hi^LY6C^lo^ monocytes emerge in the blood after MDP treatment (8, 12). We suspect that the lack of changes in CX3CR1, CCR2, and MHC II genes after MDP treatment reported in that study resulted from quantitative averaging of the naturally underrepresented I-NCMs with other dominant NCMs (12). We used *Nr4a1^–/–^* and *Nod2^–/–^* mice in conjunction with newly generated *Nr4a1^–/–^ Nod2^–/–^* double-knockout mice to identify I-NCMs as transcriptionally and functionally distinct from N-NCMs. In resting WT mice, we found that NR4A1-dependent LY6C^lo^ N-NCMs are prevalent, but there was also a small population of I-NCMs in resting *Nr4a1^–/–^* mice with cell-surface markers like those of I-NCMs induced in response to MDP. These findings are consistent with a model in which the overwhelming majority of LY6C^lo^ monocytes are NR4A1 dependent N-NCMs but a distinct population of I-NCMs is induced in response to NOD2 signaling.

We found that NOD2-dependent LY6C^lo^ I-NCMs promoted tumor immunity more potently than their N-NCM counterparts. Their migration into metastatic tumor colonies required signaling between CCL2 and CCR2, and their antitumor effects required CCL6-dependent recruitment of NK cells. CCR2 is critical for monocyte extravasation and migration (52, 53) and is needed for cell migration in models of cancer (54, 55). Intriguingly, NR4A1-dependent LY6C^lo^ NCMs migrate toward tumor colonies using the CX3CR1/CX3CL1 axis, where they have been suggested to mediate tumor lysis (56). However, they largely remain intravascular and do not commonly extravasate, potentially limiting their efficacy against pulmonary metastasis, which predominantly engrafts in the extravascular space. Hence, the effectiveness of NOD2-dependent LY6C^lo^ I-NCMs may be related to their ability to migrate into tumors in both the intra- and extravascular spaces, where they recruit NK cells that affect tumor lysis. Indeed, we found that melanoma metastasis had high expression of CCL2, which increased following lung engraftment. High expression of migration-related genes (such as *S100a4*/*6*/*10*/*11*, *Ccr2*, *Mmp12*, and *Fgfr1*) and low expression of adhesion-related genes (such as *Cx3cr1*, *Cd11a*, and *Itgal*) in NOD2–LY6C^lo^ I-NCMs suggest that they may be more adept in navigating to the sites of tumors present in different anatomic sites.

Although MDP is not available for clinical use, the liposome-encapsulated MDP analog MTP (L-MTP-PE, or mifamurtide) has been considered for the treatment of osteosarcoma (57, 58). Numerous analogs of MDP and NOD2 agonists have been developed, including targeted approaches such as liposome encapsulation (59). We found that similar to MDP, L-MTP-PE promotes the conversion of LY6C^hi^ CMs into NOD2-dependent LY6C^lo^ NCMs, suggesting its potential use in treating metastasis. Our data offer a mechanistic explanation for prior reports (57, 58) of the efficacy of MDP in a murine model of osteosarcoma, particularly as the investigators found that MDP was ineffective in *Nod2^–/–^* and *Ccr2^–/–^* mice.

Our studies also explain why subcutaneous sites of B16 melanoma are more resistant to NK cell infiltration (60). Indeed, we observed that MDP treatment was ineffective in suppressing the progression of subcutaneously injected B16F10 tumors in a mouse model, possibly since the sites were avascular. Hence, while the expansion of NOD2-dependent I-NCMs is an attractive strategy, as they can migrate into metastasis throughout the body, our data suggest that their function is dependent on signaling by CCL2/CCR2, which could vary depending on tissues, tumor burden, acquisition of CCL2 mutations, and other biological factors. This also possibly explains why the MDP analog was found to be less effective in treating pulmonary osteosarcoma metastasis when the tumor burden was high (61). The efficacy of I-NCMs was preserved even when T and B lymphocytes were genetically or pharmacologically depleted. Given that resistance to immune checkpoint inhibitors is a growing problem, our data suggest that I-NCMs might be effective in patients who progress on such therapy.

In conclusion, we described the presence of 2 distinct lineages of LY6C^lo^ NCMs. Inducible LY6C^lo^ CCR2-expressing NCMs could be expanded through the activation of NOD2 signaling to promote immunity against tumor metastasis independent of T and B lymphocytes. Given that NOD2 agonists have been approved for human use, our findings have potential clinical implications for patients with cancers resistant to current immunotherapies.

## Methods

### Sex as a biological variable

Sex was not considered as a biological variable in this study. Both male and female mice were used.

### Mice

WT CD45.2 C57BL/6J (B6) (catalog 000664) and BALB/c mice (catalog 000651), CD45.1 BALB/c mice (catalog 006584), *Nr4a1^–/–^* mice (catalog 006187), *Nod2^–/–^* mice (catalog 005763), *Cd274^–/–^* mice (catalog 032234-JAX), *Ccr2^RFP/RFP^* mice (catalog 017586), and NOD/SCID (catalog 001303), *Rag1^–/–^* (catalog 002216), and mT/mG mice (catalog 007676) were obtained from the Jackson Laboratory. The *Ccr2^RFP/+^* mice were generated by breeding *Ccr2^RFP/RFP^* mice with WT B6 mice. The mT/mG:*Nr4a1^–/–^* mice were generated by crossing mT/mG mice with *Nr4a1^–/–^* mice. *Nr4a1^–/–^* *Nod2^–/–^* mice were generated by crossing *Nr4a1^–/–^* mice with *Nod2^–/–^* mice. The *Ccr2^cre^* mice were generated by our research group. TLR7-floxed mice were obtained from Tim D. Sparwasser (Johannes Gutenberg University, Mainz, Germany). The *Ccr2^cre^ Tlr7^fl/fl^* mice were generated by crossing *Ccr2^cre^* mice with *Tlr7^fl/fl^* mice. The mice were housed at the Northwestern University Animal Care Center following standard protocols.

### Compounds

MDP (catalog tlrl-mdp, InvivoGen), MDP control (tlrl-mdpcl, InvivoGen), or L-MTP-PE sodium (HY-13682B, MedChemExpress) was administered to mice by i.v. injection at a dose of 10–20 mg/kg mouse body weight or the indicated dose. The TLR7/8 agonist R848 (vac-r848, Invitrogen), Notch signaling inhibitors DAPT (208255-80-5, Cayman Chemical) and gliotoxin (G9893, MilliporeSigma), and LPS (L2630, MilliporeSigma) were administered to mice by i.v. injection at doses of 50 μg/mouse, 25 mg/kg and 10 mg/kg, and 1 μg/mouse, respectively.

### Cancer cells

3 × 10^5^ (or the indicated number of cells) B16F10-LUC2 (catalog CRL-6475-LUC2, ATCC), B16F10-EGFP (CL053-STAN, Imanis Life Science), 4T1-LUC2-GFP or CT26-LUC2-GFP (SL020 and SL021, GeneCopoeia), or LL/2-LUC2 (ATCC) cells were injected into the mice by retro-orbital or i.v. tail vein injection.

### mRNA isolation and qPCR

Total RNA was isolated using the miRNeasy Mini Kit (QIAGEN). cDNA synthesis and qPCR were conducted as described previously (62). The primers used for *Nr4a1*, *Cx3cr1*, *Ccr2*, *Ccl2*, *Ccl6*, *Ccl9*, *Cd74*, and *C1qb* are listed in Supplemental Table 4.

### Treatment with synthetic ssRNA and viral ssRNA

#### For synthetic ssRNA.

Bone marrow monocytes were isolated from *Nod2^–/–^* or *Nr4a1^–/–^* mice using a Monocyte Isolation Kit (BM) (catalog 130-100-629, Miltenyi Biotec). Cells were treated for 36 hours with 5 μg/mL synthetic ssRNA complexed with cationic lipid (ssRNA40/LyoVec; tlrl-lrna40, Invivogen). Cells were treated with 25 μg/mL MDP for 36 hours and used as control. NCMs were determined by flow cytometry.

#### For viral ssRNA.

Influenza A virus (IAV, A/WSN/33 [H1N1]; provided by Robert Lamb, Northwestern University) and SARS-related coronavirus 2 virus (isolate USA-WA1/2020, ATCC) were expanded in MDCK cells (catalog CCL-34, ATCC) and Vero cells (CCL-81, ATCC), respectively. The virus particles in the supernatant were concentrated using Amicon Ultra-4 Centrifugal Filter Units (UFC805024, Merck Millipore). The ssRNA genome was isolated from the viral particles using a PureLink Viral RNA/DNA Mini Kit (12280050, Invitrogen). Monocyte cells were transfected for 18 hours with 6 μg/mL IAV ssRNA or 1.5 μg/mL COVID ssRNA using DOTAP Liposomal Transfection Reagent (11202375001, Roche). The NCMs were determined by flow cytometry.

### Metastatic and orthotopic tumor mouse model

For the metastatic tumor mouse model, 3 × 10^5^ B16F10-LUC2, B16F10-EGFP, 4T1-LUC2-GFP, CT26-LUC2-GFP or LL/2-LUC2 cells were injected into 6- to 8-week-old male or female mice by retro-orbital injection or tail vein injection. For the subcutaneous melanoma mouse model, 6 × 10^4^ B16F10-LUC2 cells were subcutaneously injected into 6- to 8-week-old male or female *Nr4a1^–/–^* mice in the left flank. In the subcutaneous melanoma mouse model, 3 days after subcutaneous injection of B16F10-LUC2 cells, MDP was i.v. injected (10 mg/kg) into the mice every 2–3 days for up to 2 weeks, luciferase activity was detected by LAGO every 2–3 days, body weight was checked every 4 days, and tumor size was determined by measuring the isolated B16F10 tumors using a digital caliper and calculated using the following formula: *V* = 4/3 × *π* × (*a*/2) × (*b*/2) × (*c*/2), where a, b, and c are the measured tumor length, width, and thickness, respectively.

### CCL6, CCL9, NK cells, CD4^+^ T cell, and CD8^+^ T cell depletion experiment

Purified InVivoMAb anti–mouse NK1.1 antibody (catalog BE0036; clone: PK136), InVivoMAb rat IgG2a isotype control (BE0089; clone: 2A3), InVivoMAb anti–mouse CD4 (BE0003-1; clone: GK1.5), InVivoMAb anti–mouse CD4 (BE0061; clone: 2.43), and InVivoMAb rat IgG2b isotype control (BE0090; clone: LTF-2) were purchased from BioXCell. Anti–mouse CCL6/C10 antibody (AB-487-NA), anti-Mouse CCL9/10/MIP-1 gamma Antibody (AF463), and goat IgG isotype control (AB-108-C) were purchased from R&D Systems. 8 μg/mouse for CCL9 antibody (based on the manufacturer’s instructions for the neutralization dose) or 200 μg/mouse of other of the above antibodies or isotype controls were administrated by retro-orbital injection to deplete CCL6, CCL9, NK cells, CD4^+^ T cells, or CD8^+^ T cells.

### Human blood monocyte isolation and in vitro MDP or L-MTP-PE treatment

Human blood samples were obtained from donors for lung transplantation at Northwestern Memorial Hospital in accordance with Northwestern University Institutional Review Board policies and regulations. The blood was diluted in PBS containing 5 mM EDTA (1:2 dilution), and monocytes were isolated by density gradient centrifugation on Ficoll-Paque Plus (catalog o17-1440-03, GE Healthcare) as previously described (63, 64). The CMs were further enriched by CD14^pos^ selection using CD14 MicroBeads (130-050-201, Miltenyi Biotec). Purified CD14^pos^ CMs (~1 × 10^6^ cells) were cultured in 6-well plates in complete RPMI 1640 cell culture medium containing 10% FBS and treated with MDP or L-MTP-PE for 18 or 36 hours. The human blood monocyte subsets were determined by flow cytometry based on a reported gating strategy (65) with modification for the MDP-inducible NOD2-dependent LY6C^lo^ monocyte subset.

### Flow cytometry analysis and cell sorting

Murine tissues were processed for flow cytometry analysis or adoptive transfer as described previously (66–68). Blood monocytes were sorted from MDP-treated or control B6 WT, *Nod2^–/–^*, and *Nr4a1^–/–^* mice; blood PD-L1^pos^ and PD-L1^neg^ LY6C^lo^ NCMs were sorted from B6 WT mice; and the blood samples were mixed from multiple (*n* = 5–10) mice in each group, with 3–5 repeats performed for the following RNA extraction and sequencing. RFP-labeled LY6C^lo^ NCMs were sorted from MDP-treated mT/mG:*Nr4a1^–/–^* mouse blood or spleen. All antibodies used for flow cytometry (Supplemental Table 3) were purchased from BD Biosciences, BioLegend, or Thermo Fisher Scientific.

### Transcriptomics

Total RNA was extracted from mouse blood monocytes using an Arcturus PicoPure RNA Isolation Kit (catalog KIT0204, Thermo Fisher Scientific) or RNeasy Plus Micro Kit (74034, QIAGEN). Qualified total RNA samples were preamplified, and cDNA synthesis was performed using the SMART-Seq v4 Ultra Low Input RNA Kit for sequencing (Clontech/Takara Bio). Library preparation, RNA-Seq, data acquisition, and principal component analysis (PCA) were performed as previously described (67). Pathway enrichment analysis was conducted using the online tool Metascape (69). Gene set enrichment analysis (GSEA) was conducted using BubbleGUM (70, 71), and gene signatures for myeloid cells for GSEA in this study were chosen based on the published literature (72).

### LAGO/in vivo imaging system (IVIS) imaging detection

Mice were injected intraperitoneally with d-luciferin (catalog 122799, PerkinElmer) at a dose of 300 μg/kg. Ten minutes after injection, bioluminescence images were captured using an IVIS Spectrum in vivo imaging system (PerkinElmer).

### Two-photon microscopy

Three days after retro-orbital injection of 3 × 10^5^ B16F10-GFP cells, 240,000 splenic LY6C^lo^ monocytes were sorted from multiple MDP-treated *Nr4a1^–/–^* mice, stained with 5 μg/mL Hoechst 33342 for 10–20 minutes at room temperature, and injected retro-orbitally into *Nod2^–/–^* mice. Mice were anesthetized for 2-photon intravital lung microscopy imaging using a Nikon A1R-MP^+^ multiphoton microscope system as described previously (68).

### TEM

Blood samples were obtained from MDP-treated *Nr4a1^–/–^* or *Nod2^–/–^* mice and mixed in each group (each group was analyzed in duplicate in the experiment). Approximately 60,000–130,000 blood LY6C^hi^ or LY6C^lo^ monocytes were sorted. The sorted monocytes were embedded in 3% low-melting-point agarose and fixed with 2.5% glutaraldehyde in 0.1 M cacodylate buffer for ultrastructural examination using an FEI Tecnai Spirit G2 TEM.

### Mouse lung dissection and IHC staining

Mouse lung tissue dissection and IHC staining were performed as previously described (73). Briefly, transcardial perfusion was performed on adult mice with cold PBS followed by 4% paraformaldehyde (PFA) after anesthesia. Mouse lungs were inflated with a 30% sucrose:OCT (1:1) mixture and then dissected, followed by postfixation in 4% PFA for 4 hours at 4°C. The lung tissue was dehydrated in 30% sucrose overnight (O/N) at 4°C, then embedded in OCT. For IHC staining, 10-μm frozen sections were washed with PBS, permeabilized with PBST (0.3% Triton in PBS), blocked with blocking buffer containing 5% donkey serum in PBST for 1 hour at room temperature, and incubated with primary antibodies (GFP: A-11122, Invitrogen, or ab13970, Abcam RFP: 5F8/5F8, Proteintech; CD31: AF3628, R&D Systems, or 553370/MEC 13.3, BD Biosciences; CCL2: NBP1-07035SS, Novus; Luciferase antibody: NB100-1677SS, Novus; CCR2: ab216863, Abcam) in blocking solution O/N at 4°C. The sections were washed with PBS and incubated with secondary antibodies (Alexa Fluor 488 Donkey anti-chicken IgY: A78948; Alexa Fluor 488 Donkey anti-Rabbit IgG: A-21206; Alexa Fluor 647 Donkey anti-Goat IgG: A-21447; Alexa Fluor 568 Donkey anti-Rabbit IgG: A10042; Alexa Fluor 405 Donkey anti-Goat IgG: A48259; Alexa Fluor 647 Goat anti-Rat IgG: A21247; Thermo Fisher Scientific) in PBS for 1 hour at room temperature. After washing with PBS, the sections were counterstained with DAPI and mounted with mounting medium. Fluorescence images were acquired using a Nikon A1 confocal laser microscope with NIS-Elements Viewer 4.20 software. Images were processed and analyzed with Adobe Photoshop and Fiji (ImageJ, NIH) software.

### In vitro and in vivo detection of uptake of B16F10 material by I-NCMs

#### In vitro detection.

RFP-labeled I-NCMs were sorted from blood or spleen from MDP-treated mT/mG:*Nr4a1^–/–^* mice. Sorted blood RFP-I-NCMs were cocultured with B16F10-GFP at a ratio of 5:1 (10,000 monocytes/2,000 tumor cells) on poly-l-lysine–coated coverslips in 24-well plates for 60 hours for immunocytochemistry (ICC). Sorted spleen RFP-I-NCMs were cocultured with B16F10-GFP at a ratio of 5:1 (50,000 monocytes/10,000 tumor cells) in a 12-well plate for up to 60 hours for constant observation of colocalization of GFP and RFP signals by confocal microscopy and flow cytometry confirmation. Three wells of cells were pooled at 60 hours after coculture for detection of GFP-positive monocytes with intracellular staining of GFP antibody by flow cytometry.

#### In vivo detection.

*Nr4a1^–/–^* mice were continuously treated with MDP for 6 days. A total of 5 × 10^5^ B16F10-GFP cells were injected by retro-orbital injection on day 2 and day 5 after injection of MDP to ensure there was a sufficient number of B16F10-GFP cells in both blood vessel and lung tissues. Blood samples were taken from the facial vein or heart at 18 or 36 hours, and lung samples were taken at 36 hours after the second dose of B16F10-GFP cells and prepared for the detection of GFP signals with intracellular staining of GFP antibodies in gated monocyte subsets by flow cytometry.

### Direct tumor cytotoxicity of monocytes, eosinophils, alveolar macrophages, and interstitial macrophages

LY6C^hi^ monocytes and LY6C^lo^ monocytes were sorted from MDP-treated *Nr4a1^–/–^* or *Nod2^–/–^* mice and treated with or without IFN-γ (20 ng/mL) and LPS (100 ng/mL) for 24 hours, followed by coculture with B16F10-LUC2 cells at a ratio of 50:1 (25,000 monocytes/500 B16 cells per well) in a 96-well plate for 24 hours. Eosinophils, alveolar macrophages, and interstitial macrophages were sorted from lungs of WT B6 mice and cocultured with B16F10-LUC2 cells at ratio of 6:1 (9,000 eosinophils, alveolar macrophages, or interstitial macrophages/1,500 B16 cells per well) in 96-well plates for 24 hours. Cell proliferation and apoptosis were determined by assaying luciferase activity using a Pierce Firefly Luciferase Glow Assay Kit (catalog 16176, Thermo Fisher Scientific) according to the manufacturer’s protocol.

### Statistics

Data were collected from at least 3 repeats in each group and are presented as mean ± SEM. All statistical calculations were performed using GraphPad Prism v10.2.3. For comparison of the means of 2 population groups, 2-tailed *t* test was used; for comparison of the means of multiple (>2) population groups, Kruskal-Wallis test (nonparametric test), or 1-way ANOVA (including Brown-Forsythe’s and Welch’s ANOVA) was used. *P* values of less than 0.05 were considered significant.

### Study approval

All animal protocols and procedures were approved by the Institutional Animal Care and Use Committee (IS00008524, IS00015233, IS00010791) at Northwestern University. Animals received humane care in compliance with the NIH *Guide for the Care and Use of Laboratory Animals* (National Academies Press, 2011) and the Principles of Laboratory Animal Care formulated by the National Society for Medical Research. Human study protocols were approved by the IRB at Northwestern University Feinberg School of Medicine (STU00106589). All study subjects provided written informed consent prior to participation in the study.

### Data availability

The generated bulk RNA-Seq datasets for the mouse blood monocytes are available in the NCBI Gene Expression Omnibus repository (GEO GSE277303). The mouse lung transplantation scRNA-Seq datasets can be found in the NCBI Gene Expression Omnibus repository (GSE166679). The unpublished spleen transplantation scRNA-Seq datasets are available from the corresponding author upon request. Other datasets are available in the Supporting Data Values file.

## Author contributions

XL and AB conceptualized the study, designed the experiments, interpreted the data, and wrote the manuscript. FLNS performed 2-photon imaging, and CT provided technical support for IHC and ICC experiments and data analysis. HAV performed RNA-Seq. ZR and YY analyzed bulk and scRNA-Seq data. QW conducted lung transplantation. WY and TT contributed to sample preparation for lung transplantation or spleen transplantation for single-cell sequencing. MM conducted qPCR. SMCM and EL prepared COVID-19 and IAV ssRNA. XL, HS, MEK, and EL were involved in conducting murine and cell culture experiments, manipulating mouse breeding, or optimizing experimental conditions. EJC, MEA, RG, HP, NSC, and GRSB contributed to the data interpretation and manuscript editing and revision. LJH provided the *Ccr2^cre^* mice. All authors critically reviewed the data and approved the manuscript.

## Supplementary Material

Supplemental data

Supplemental table 1

Supplemental table 2

Supplemental table 3

Supplemental table 4

Supplemental video 1

Supporting data values

## Version 1 11/15/2024

Electronic publication
