# Supplementary material for: Inducible CCR2+ nonclassical monocytes mediate the regression of cancer metastasis
Source: J Clin Invest. 2024 Oct 1;134(22):e179527. doi: 10.1172/JCI179527 (PMC11563681; doi:10.1172/JCI179527)
Supplement: Supplemental data [file jci-134-179527-s161.pdf]

Supplementary Materials for

Inducible CCR2+ nonclassical monocytes mediate the regression of cancer metastasis

Xianpeng Liu,[1] Ziyou Ren,[2] Can Tan,[3] Félix L. Núñez-Santana,[1] Megan E. Kelly,[1] Yuanqing Yan,[1] Haiying Sun,[1] Hiam Abdala-Valencia,[4] Wenbin Yang,[1] Qiang Wu,[1] Takahide Toyoda,[1] Marija Milisav,[1] S. Marina Casalino-Matsuda,[4] Emilia Lecuona,[1] Emily Jeong Cerier,[1] Lena J. Heung,[5] Mohamed E. Abazeed,[6] Harris Perlman,[7] Ruli Gao,[8] Navdeep S. Chandel,[4] G.R. Scott Budinger,[4] and Ankit Bharat[1,4,*]

[1]Division of Thoracic Surgery/Canning Thoracic Institute, Feinberg School of Medicine, Northwestern University/Northwestern Medicine, Chicago, Illinois, USA. [2]Department of Dermatology, [3]Division of Cardiology, Department of Medicine, and [4]Pulmonary and Critical Care Medicine, Feinberg School of Medicine, Northwestern University, Chicago, Illinois, USA. [5]Division of Infectious Diseases, Department of Medicine, Cedars-Sinai Medical Center, Los Angeles, California, USA. [6]Department of Radiation Oncology, [7]Division of Rheumatology, and [8]Department of Biochemistry, Feinberg School of Medicine, Northwestern University, Chicago, Illinois, USA.

*Corresponding Author: Ankit Bharat MD, FACS; Email: ankit.bharat@nm.org

This PDF file includes:

Supplemental Figures and Legends for Fig. S1-S20

Legends for Table S1-S4

Legend for Movie S1

Supplemental Fig.1

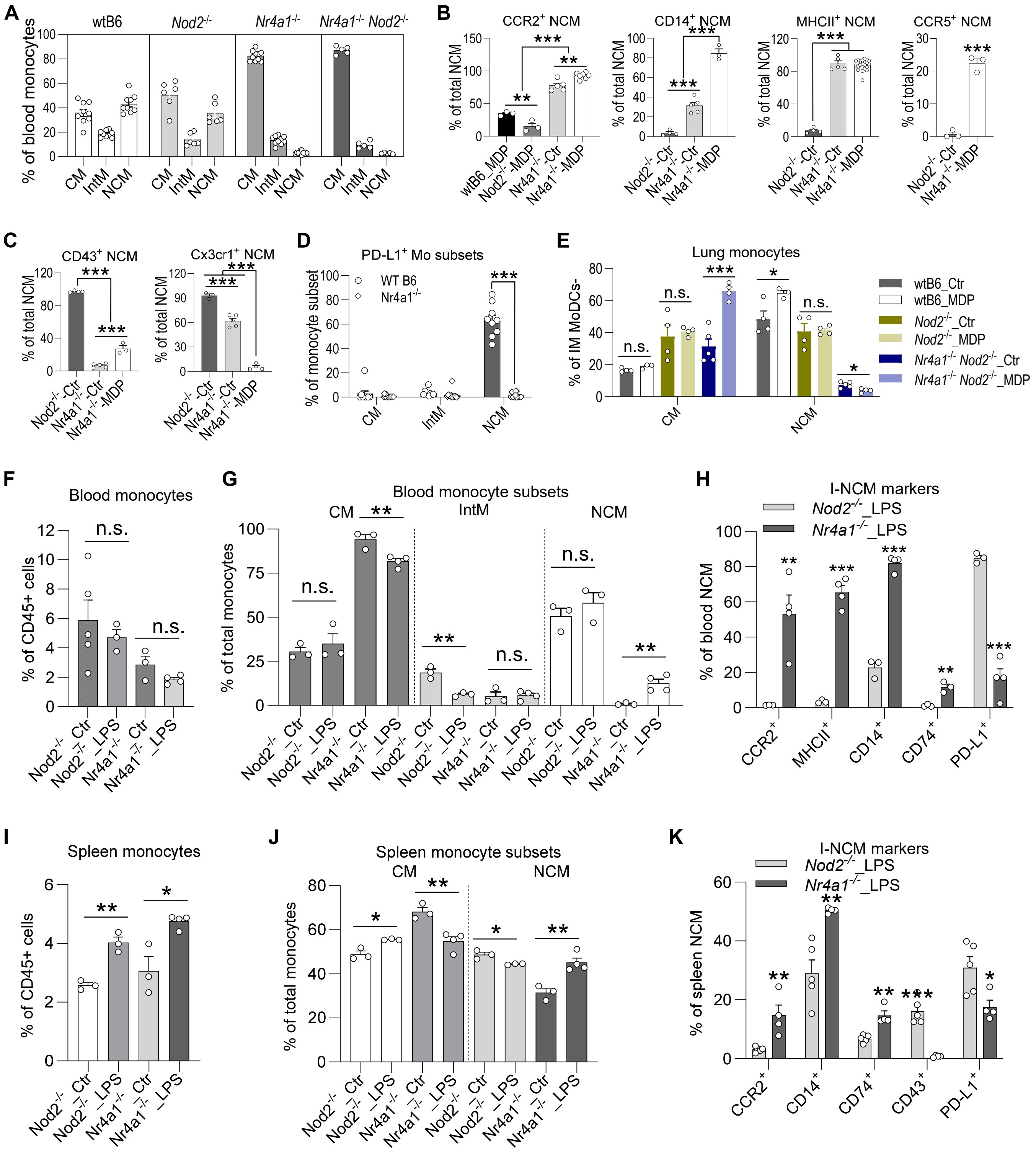

Fig.S1. <u>*I-NCMs are underrepresentation in steady state but increase following LPS-treatment-induced inflammatory stimuli.*</u>

(**A**) Evaluation of blood monocytes in steady state in wild-type and mutant mice (n=5-10). For quantification of NCM, we used total LY6C^{lo} monocytes.

(**B**-**D**) Verification of the selected LY6C^{lo} NCM gene signatures by flow cytometry in wild-type, *Nod2*$^{-/-}$ and *Nr4a1*$^{-/-}$ mice in steady state or after MDP treatment. (**B**-**C**) Verification of the selected I-NCM gene signatures by flow cytometry. (**D**) PD-L1 marks blood N-NCMs, but not I-NCMs. Flow cytometry data indicating the differential expression of PD-L1 in monocyte subsets.

(**E**) The ratio of monocyte subsets detected by flow cytometry in lung in the indicated mice in steady state or after MDP treatment.

(**F**-**K**) LPS-treatment induces I-NCM population in blood and spleen in *Nr4a1*$^{-/-}$ mice. (**F**-**G**) Detection the ratio of monocytes in CD45+ cells or monocyte subsets in total blood monocytes in LPS-treated or nontreated *Nod2*$^{-/-}$ and *Nr4a1*$^{-/-}$ mice. (**H**) Comparison of the expression of the selected I-NCM markers in blood NCM in LPS-treated *Nod2*$^{-/-}$ and *Nr4a1*$^{-/-}$ mice. (**I**-**J**) Detection of the ratio of monocytes in CD45+ cells or monocyte subsets in total splenic monocytes in LPS-treated or nontreated *Nod2*$^{-/-}$ and *Nr4a1*$^{-/-}$ mice. (**K**) Comparison of the expression of the selected I-NCM markers in splenic NCM in LPS-treated *Nod2*$^{-/-}$ and *Nr4a1*$^{-/-}$ mice. *Nod2*$^{-/-}$ or *Nr4a1*$^{-/-}$ mice were treated with LPS by I.V. injection (1μg/mouse). At 36h post LPS injection, the blood and spleen were collected and prepared for flow cytometry.

Data are presented as Mean ± SEM; n=3-20 in each group; $*p < 0.05$, $**p < 0.01$, $***p < 0.001$; 1-way ANOVA test was used for (**B**-**C**), 2-tailed t test for (**D**-**K**).

Supplemental Fig.2

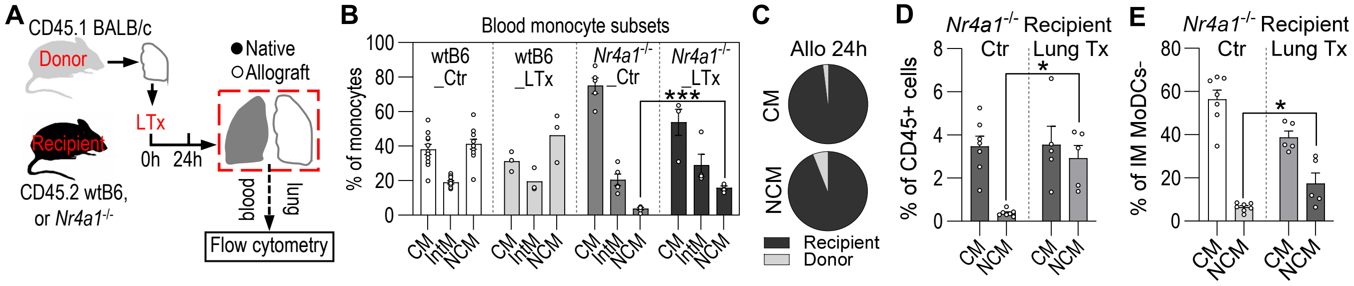

F

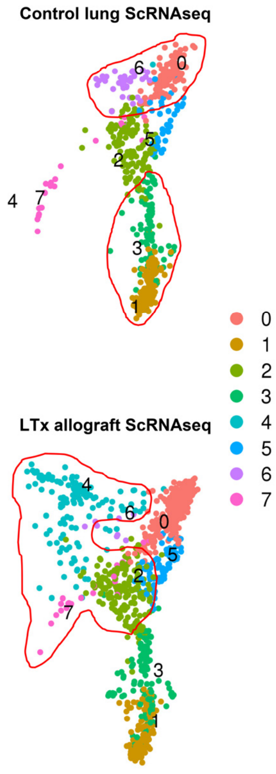

wtB6_LTx 24h: CM ↓ (C6 C0 C5) I-NCM ↓ (C4 C7 C2) N-NCM ↓ (C1 C3)

	C6	C0	C5	C4	C7	C2	C1	C3
Ly6c1	x							
Ly6c2	x	x						
Sell	x	x						
Fos	x	x	x					
Lyz2	x	x	x					
Fn1	x	x	x	x				
Cd177	x	x	x					
	C6	C0	C5	C4	C7	C2	C1	C3
Nr4a1							x	x
Cx3cr1							x	x
Klf2							x	x
Klf4							x	x
Cebpb							x	x
Pou2f2							x	x
Itgal							x	x
Cd36							x	x
Fcgr4							x	x
Cd274							x	
Itgax							x	x
Pparg							x	
	C6	C0	C5	C4	C7	C2	C1	C3
Ccr2	x	x	x		x	x		
Cd14				x				
Cebpa	x	x	x					
Cd209a					x			
Irf4					x			
C1qb				x				
C1qc				x				
Myc				x				
Batf3	x					x		
Id3					x			
Cd74					x	x		
H2-Aa					x	x		
H2-Ab1					x	x		
H2-Dma				x	x	x		
H2-DMb1				x	x	x		
H2-Eb1					x	x		
Ccl6		x		x				
Ccl9	x	x		x				
Ccr1				x				
Ccr5				x				
Ipl				x				
Fcgr2b				x		x		
Cd16				x		x		
Ciita						x		
Slamf7					x			
Slamf8				x		x		

G

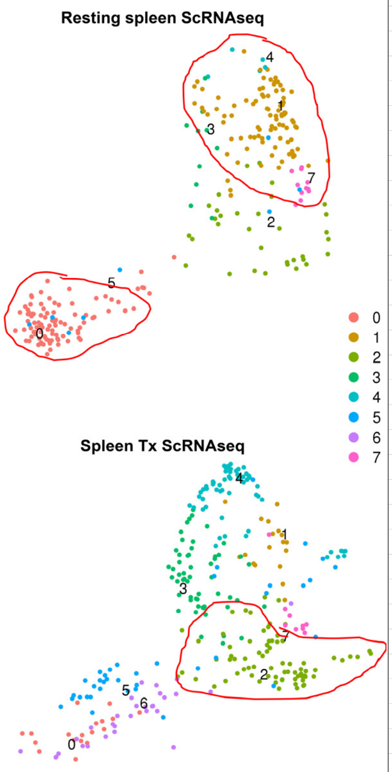

wtB6_spleen Tx 24h: CM ↓ (C1 C4 C7) I-NCM ↓ (C2) N-NCM ↓ (C0)

	C1	C4	C7	C3	C2	C6	C5	C0
Ly6c2	x	x	x					
Sell	x	x	x					
Fos		x						
Lyz2	x							
Fn1	x	x						
Cd177	x	x						
	C1	C4	C7	C3	C2	C6	C5	C0
Nr4a1								x
Cx3cr1								x
Klf2								x
Klf4								x
Cebpb								x
Pou2f2						x		x
Itgal								x
Cd36								x
Fcgr4						x		x
Cd274								
Itgax								x
Pparg								x
	C1	C4	C7	C3	C2	C6	C5	C0
Ccr2	x				x			
Cd14		x						
Cebpa	x							
C1qa					x			
C1qb					x			
C1qc					x			
Batf3					x			
Cd74					x			
H2-Aa					x			
H2-Ab1					x			
H2-Dma	x				x			
H2-DMb1					x			
H2-Eb1					x			
Ccl6	x	x						
Ccl9	x	x	x					
Ccr1		x		x				
Ccr5		x						
Fcgr2b					x	x		
Ciita					x			
Slamf7								
Slamf8					x			

Fig.S2. *I-NCMs increase following solid organ transplant.*

(**A**-**E**) Transplantation- induced or augmented NR4A1-independent I-NCM subsets in blood, lung and spleen.

(**A**). Experimental schematic showing allogeneic lung transplantation (LTx).

(**B**) The ratio of monocyte subsets in total monocytes detected by flow cytometry in blood in the indicated control or recipient mice 24h post LTx. Data are presented as Mean ± SEM; n=4-5 in each group; *Nr4a1*$^{-/-}$_Ctr_NCM vs. *Nr4a1*$^{-/-}$_LTx_NCM, ***$p < 0.001$; two-tailed t test.

(**C**) The origin of the repopulated CM or NCM in the perfused donor lung at 24h post LTx (CD45.1 BALB/c to CD45.2 wtB6 mice).

(**D**-**E**) The ratio of monocytes or monocyte subsets in allograft lung CD45+ cells (**D**) or in total monocytes (**E**), respectively. Ctr: *Nr4a1*$^{-/-}$ lung; LTx: CD45.1 BALB/c to CD45.2 *Nr4a1*$^{-/-}$ mice). Data are presented as Mean ± SEM; n=5-7 in each group; Ctr_NCM vs. LTx_NCM, *$p < 0.05$; 2-tailed t test.

(**F**-**G**) CM, N-NCM, and I-NCM were characterized by secondary analysis of scRNAseq data from allogeneic lung LTx allograft lung (**F**) or syngeneic spleen Tx recipients (**G**) and are indicated by red line. The typical markers specific for CM, N-NCM, and I-NCM in lung or spleen are illustrated. C=cluster.

Supplemental Fig.3

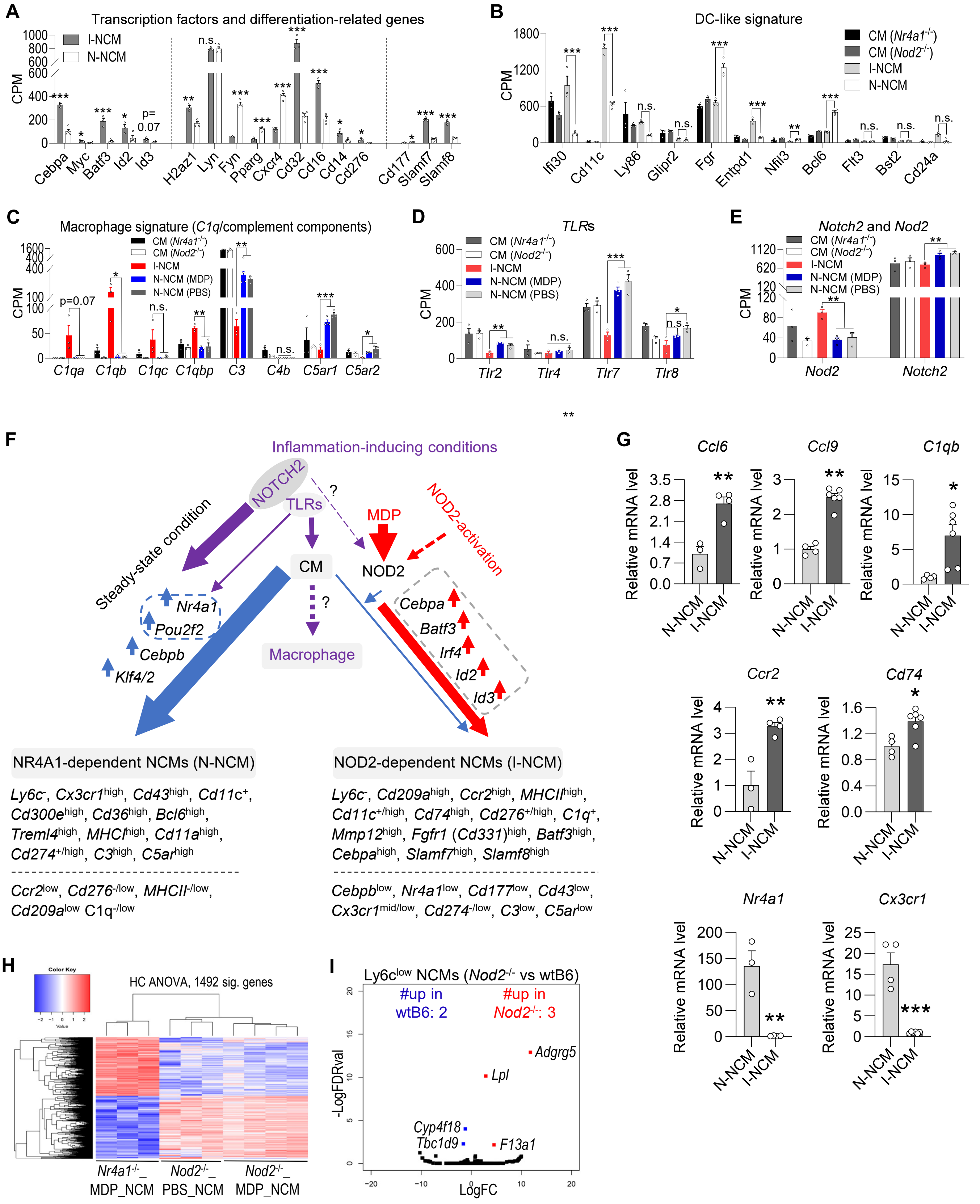

Fig.S3. *Transcriptomic profiling of I-NCM and N-NCM.*

Blood samples were taken from facial vein from MDP-treated *Nr4a1*$^{-/-}$ or *Nod2*$^{-/-}$ mice and used for RNA extraction and subsequent bulk RNA sequencing.

(**A**-**E**) Pairwise comparison of gene expression of the selected transcriptional factors (**A**, left), differentiation-related genes (**A**, middle and right), cDC-like genes (**B**), complement components genes (**C**), TLR genes (**D**), *Nod2* and *Notch2* genes (**E**) in blood monocyte subsets in MDP-treated or untreated *Nod2*$^{-/-}$ mice and *Nr4a1*$^{-/-}$ mice. RNAseq CPM data are presented as the mean ± SEM; n=3-4 in each group; *p < 0.05, **p < 0.01, ***p < 0.001; 1-way ANOVA test.

(**F**) Postulated differentiation pattern of CM into N-NCM and I-NCM with typical gene signatures for each subset.

(**G**) Verification of the selected NCM markers using qPCR. The total RNA was extracted from sorted splenic NCM from *Nod2*$^{-/-}$ mice or MDP-treated *Nr4a1*$^{-/-}$ mice. The mRNA expression of the selected NCM markers was determined by qPCR. At least 3 repeats were performed, and each sample were technically duplicated, data are presented as Mean ± SEM; *p < 0.05, **p < 0.01, ***p < 0.001; 2-tailed t-test.

(**H**) Heatmap analysis of DEGs in LY6C^{lo} monocytes in control *Nod2*$^{-/-}$ mice and MDP-treated *Nr4a1*$^{-/-}$ mice or *Nod2*$^{-/-}$ mice.

(**I**) Volcano plot shows top DEGs in LY6C^{lo} NCMs in *Nod2*$^{-/-}$ mice and wtB6 mice.

Supplemental Fig.4

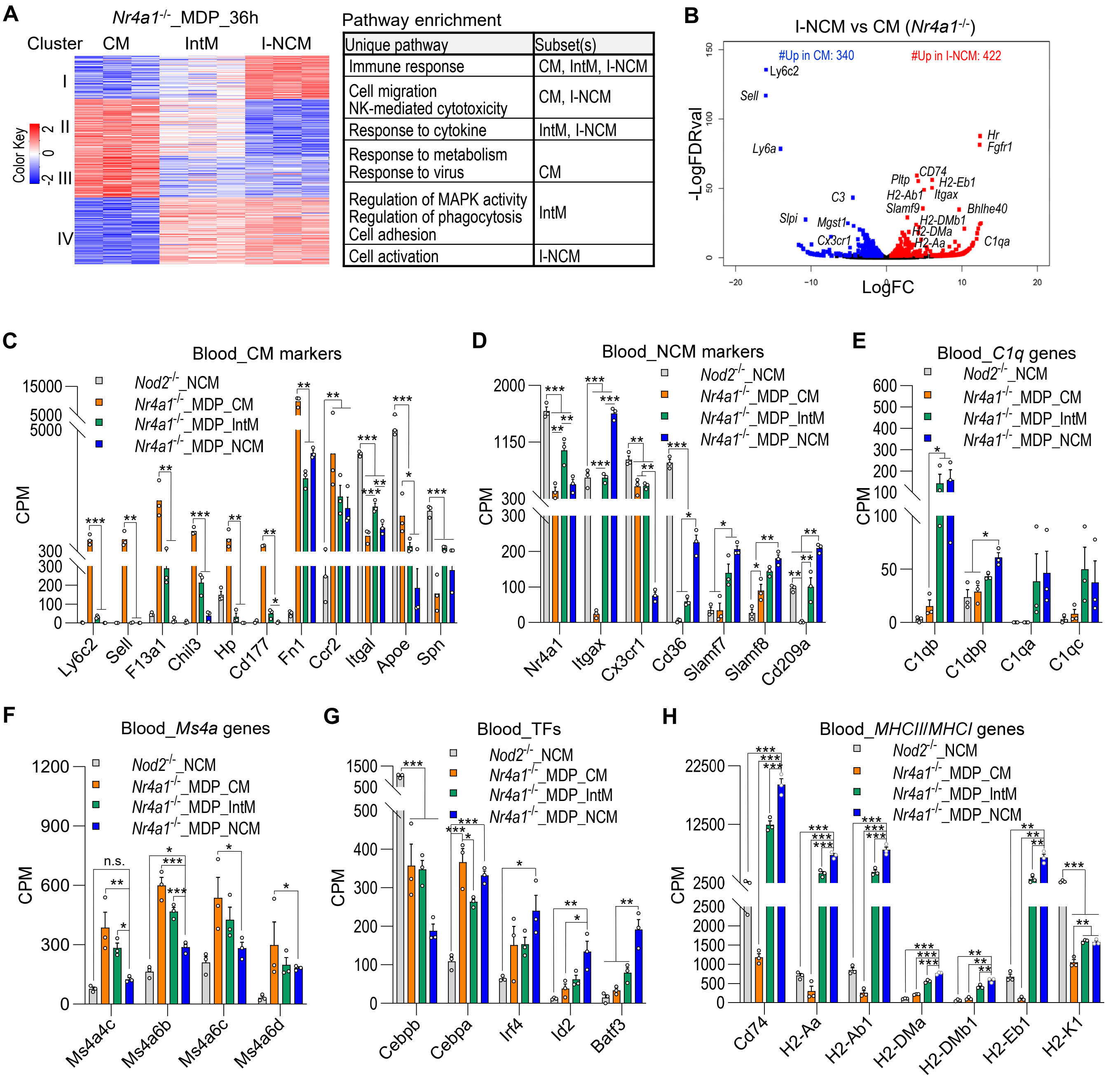

Unique pathway	Subset(s)
Immune response	CM, IntM, I-NCM
Cell migration<br>NK-mediated cytotoxicity	CM, I-NCM
Response to cytokine	IntM, I-NCM
Response to metabolism<br>Response to virus	CM
Regulation of MAPK activity<br>Regulation of phagocytosis<br>Cell adhesion	IntM
Cell activation	I-NCM

I

For IntM (from Mo clusters):
Ly6c2, *Cx3cr1*, *Sell*, *Nr4a1*
Cebpb, *Cd177*:

intermediate level (vs CM and NCM)

For CD209a⁺ IntM/I-NCM (from IntM culsters):
Ccr2, *MHCII*, *Cd74*, *Cebpa*, *Ciita*, *Irf4*, *Batf3*, *Cd209a*, *Slamf7*, *C1qbp*, *C1qb*, *Ms4a4c*, *Ms4a6b/c/d*: +/high

Cd36, *Sell*, *Treml4*, *Itgal*, *F13a1*, *chil3*, *Hp*: low/-
(vs other clusters in IntM clusters)

For I-NCM (from CD209a⁺ IntM/I-NCM cluster):
Ccr2, *MHCII* (*H2-Aa/Ab1/Dma/Eb1*), *Cd74*, *Cebpa*, *Cd209a*, *Slamf7*, *Batf3*: high⁺

Cd36, *Sell*, *F13a1*, *Chil3*, *Hp*: low/-
(within **CD209a⁺** IntM/I-NCM clusters)

Fig.S4. *Transcriptional profiles of mouse blood monocyte subsets.*

Blood samples were taken from facial vein from MDP-treated or untreated *Nr4a1*$^{-/-}$ or *Nod2*$^{-/-}$ mice and were used for RNA extraction and subsequent bulk RNA sequencing.

(**A**) Heatmap analysis of blood monocyte subsets (left panel) and unique pathways enriched in each monocyte subsets (right panel) in MDP-treated *Nr4a1*$^{-/-}$ mice.

(**B**) Volcano plot demonstrates top DEGs in CMs and LY6C^{lo} NCMs in MDP-treated *Nr4a1*$^{-/-}$ mouse blood.

(**C**-**I**) Transcriptional markers for distinguishing CD209a^{+} LY6C$^{Int/lo}$ IntM and CD209^{hi} LY6C$^{lo/-}$ I-NCM clusters. (**C**-**H**) Pairwise comparison of gene expression of the selected markers in the indicated mouse blood monocyte subsets in bulk RNAseq data. RNAseq CPM data are presented as the mean ± SEM; n=3 in each group; *p < 0.05, **p < 0.01, ***p < 0.001; 1-way ANOVA test. (**I**) Strategies and potential markers that may be used for distinguishing CD209a^{+} LY6C$^{Int/lo}$ IntM and CD209^{hi} LY6C$^{lo/-}$ I-NCM clusters.

Supplemental Fig.5

A

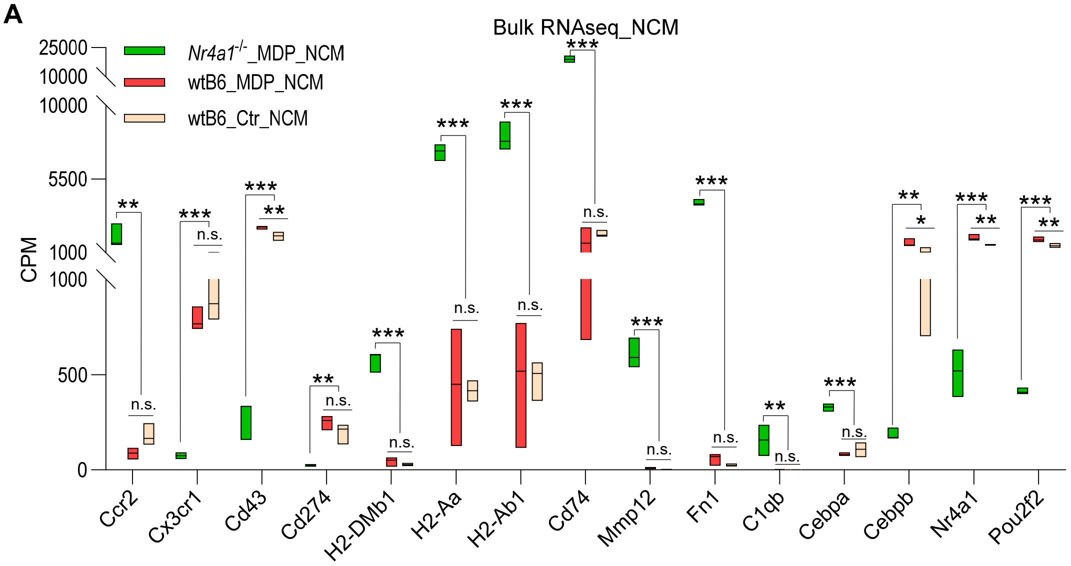

B

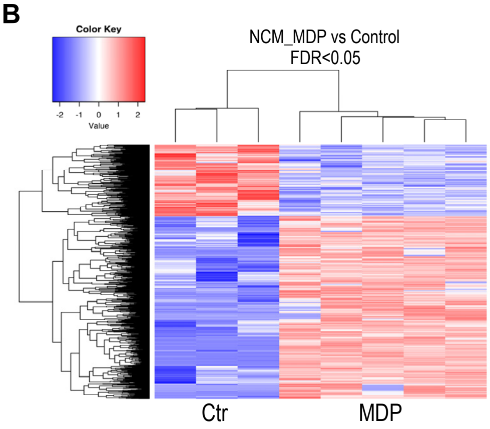

wtB6_NCM (MDP 36h vs Ctr) (up>1.5-fold, FDR<0.05)
Defense response
Mitochondrial metabolism/apoptotic signaling
Type I interferon-mediated signaling
Vascular permeability/Endothelial cell migration
Cell morphogenesis/cell adhesion
Mitotic cell cycle process

C

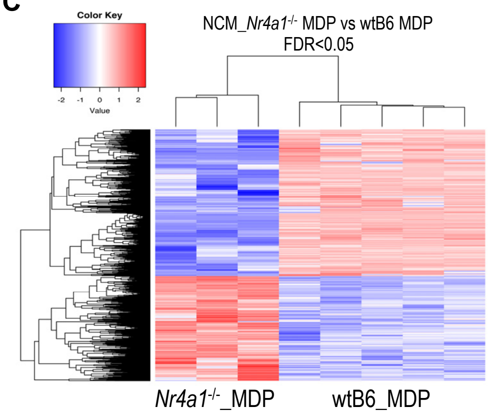

Nr4a1-/-_MDP_NCM vs wtB6_MDP_NCM (up>1.5-fold, FDR<0.05)
Adaptive immune response
Antigen proc. and pres. via MHCII
Cytokine-mediate Inflammatory response
Leukocyte activation/ differentiation/ apoptosis
Regulation of chemotaxis
Regulation of MAPK cascade

D

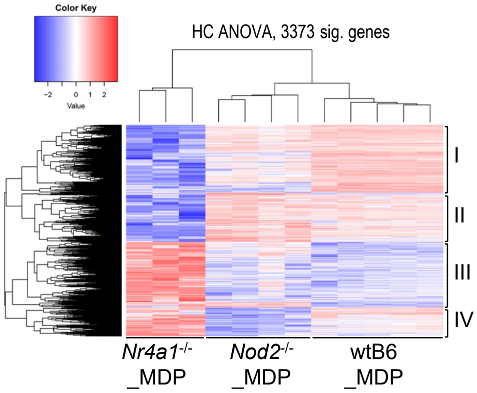

MDP_NCM (Nr4a1-/- vs Nod2-/- vs wtB6)	
I	Regulation of MAPK cascade/cell apoptosis<br>Regulation of cell migration<br>Cellular response to stress
II	Regulation of MAPK cascade/NF-kappaB<br>Response to cytokine stimulus/bacterial molecule<br>Leukocyte activation
III	Regulation of leukocyte activation/chemotaxis<br>Antigen proc, and pres. via MHC class II<br>Cellular response to interleukin-4
IV	Transport along microtubule/endocytosis<br>Cellular response to type II interferon<br>Regulation of amide metabolic process

Fig.S5. *Transcriptional profiles of wtB6 mouse blood LY6C^{lo} monocyte subsets.*

Blood samples were taken from facial vein from MDP-treated or untreated wtB6, *Nr4a1*$^{-/-}$ or *Nod2*$^{-/-}$ mice, and were used for RNA extraction and subsequent bulk RNA sequencing.

(**A**) Pairwise comparison of gene expression of the selected I-NCM markers in LY6C^{lo} monocyte in untreated wtB6 mice, MDP-treated wtB6 mice or *Nr4a1*$^{-/-}$ mice. RNAseq CPM data are presented as the mean ± SEM; n=3-5 in each group; *p < 0.05, **p < 0.01, ***p < 0.001; 1-way ANOVA test.

(**B**) Heatmap analysis of blood monocyte subsets (upper panel) and unique pathways enriched in LY6C^{lo} monocyte (lower panel) in MDP-treated or untreated wtB6 mice.

(**C**) Heatmap analysis of blood monocyte subsets (upper panel) and unique pathways enriched in LY6C^{lo} monocyte (lower panel) in MDP-treated *Nr4a1*$^{-/-}$ and wtB6 mice.

(**D**) Heatmap analysis of DEGs in LY6C^{lo} monocytes (left panel) and unique pathways enriched in LY6C^{lo} NCMs (right panel) in MDP-treated *Nr4a1*$^{-/-}$ mice, *Nod2*$^{-/-}$ mice and wtB6 mice.

Supplemental Fig.6

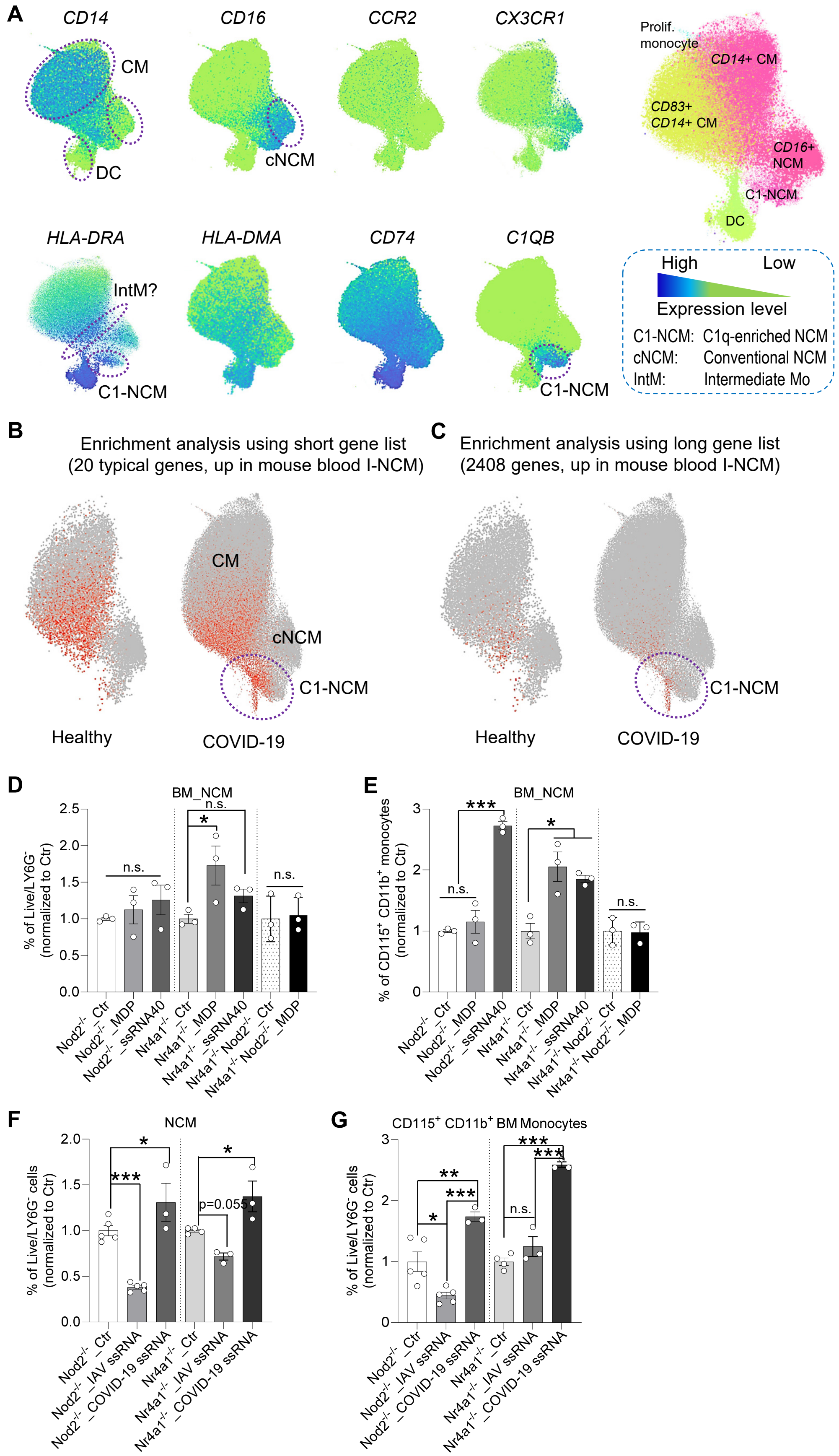

Fig.S6. *COVID-19 infection or ssRNA treatment increases I-NCM.*

(**A-C**) Transcriptional similarity between mouse blood I-NCMs and human blood C1-NCMs.

(**A**) The enrichment of blood I-NCM gene markers in human blood monocyte subsets, which is analyzed using an interactive single cell web portal (18). CM, C1q-enriched NCM cluster (C1-NCM) and conventional NCM (cNCM) are circulated with dashed blue line.

(**B**) Expression, alignment and enrichment of a short list of genes (20 genes, **Table S1**) that are upregulated in mouse blood I-NCMs were analyzed in human blood monocytes by secondary analysis of the published scRNA-seq data (18).

(**C**) Expression, alignment and enrichment of a long list of genes that are upregulated (fold-change >1.5, I-NCM vs N-NCM, 2408 genes, **Table S2**) in mouse blood I-NCMs were analyzed in human blood monocytes.

(**D**-**G**) Effects of ssRNA on differentiation of monocytes. Bone marrow monocytes were isolated from *Nod2*$^{-/-}$, *Nr4a1*$^{-/-}$ or *Nr4a1*$^{-/-}$ *Nod2*$^{-/-}$ mice and treated with MDP, synthetic ssRNA or viral ssRNA. (**D**-**E**) Cells were treated with synthetic complexed ssRNA or MDP for 36 hours, the ratio of NCM in total live/LY6G$^-$ cells (**D**) or CD115$^+$ CD11b$^+$ cells (**E**) were determined by flow cytometry. (**F**-**G**) Monocyte cells were transfected with IAV ssRNA or COVID-19 ssRNA for 18h. The ratio of NCM (**F**) or CD115$^+$ CD11b$^+$ monocytes (**G**) in total live/Ly6G$^-$ cells were determined by flow cytometry. Data are presented as Mean ± SEM; n=3-5 in each group; *$p < 0.05$, **$p < 0.01$, ***$p < 0.001$; 1-way ANOVA test (for three-column data sets) and 2-tailed t test (for two-column data sets).

Supplemental Fig.7

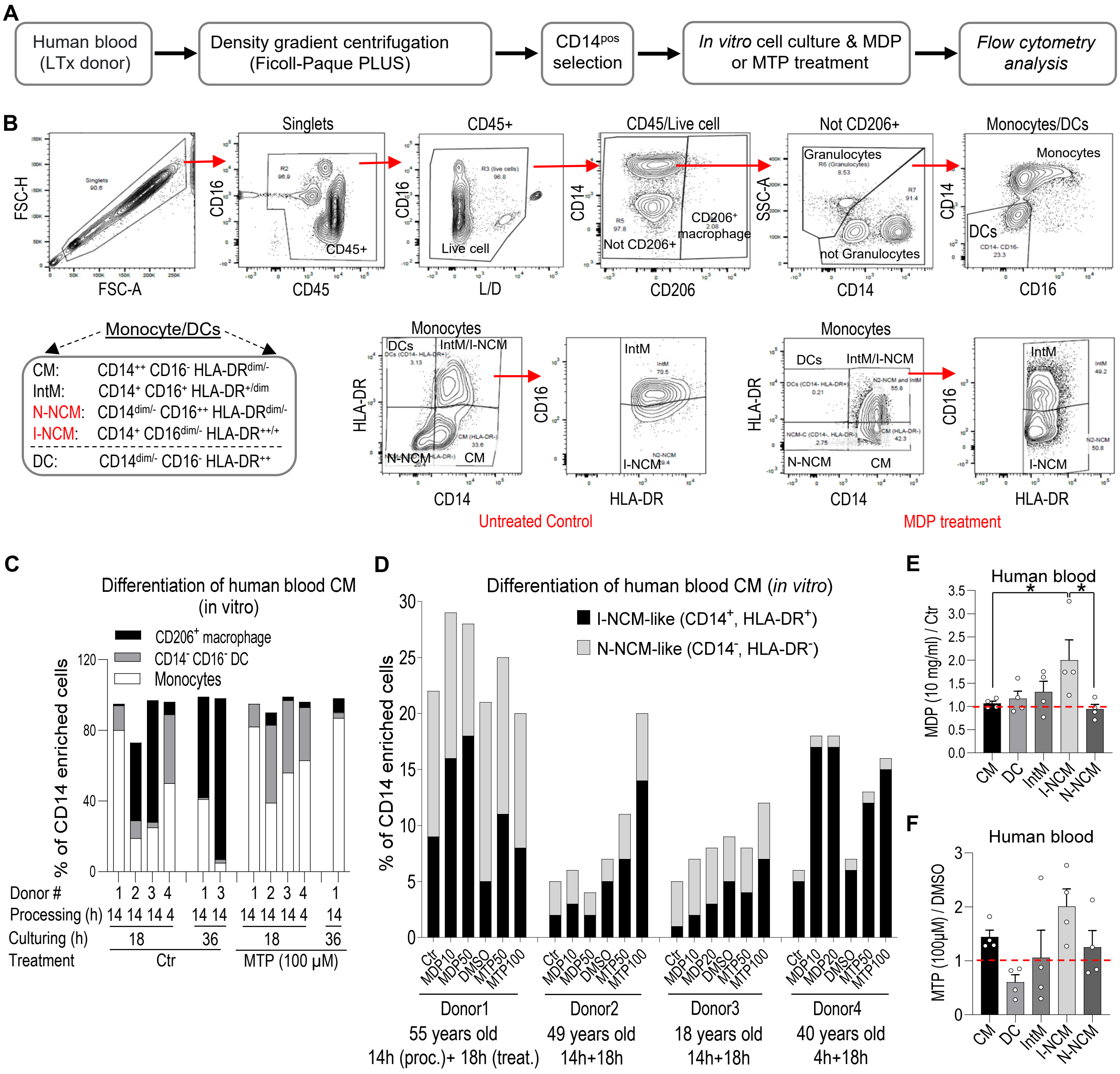

Fig.S7. *I-NCM in human blood.*

(**A**). Experimental design showing human blood sample preparation for in vitro culturing and flow cytometry analysis. (**B**). Gating strategy optimized for defining I-NCM subset in human blood. (**C**). Differentiation of CM into $CD206^+$ macrophage during in vitro culturing and MTP-treatment in individual sample was determined by flow cytometry. (**D**). I-NCM and N-NCM monocyte subsets in individual sample were analyzed by flow cytometry after MDP or MTP treatment in in vitro culturing system. Processing time (from receiving sample to starting treatment) plus treatment time are labeled for each sample in (**C**) and (**D**). (**E-F**). Normalized flow cytometry data indicating the induction of I-NCM subsets after MDP treatment (**E**) or L-MTP-PE treatment (**F**) in all 4 samples. The relative level of each cell type or subset in treatment group was calculated and normalized to that in control group (untreated control or DMSO group). Data are presented as Mean ± SEM; n=4 in each group; *$p < 0.05$; one-way ANOVA test.

Supplemental Fig.8

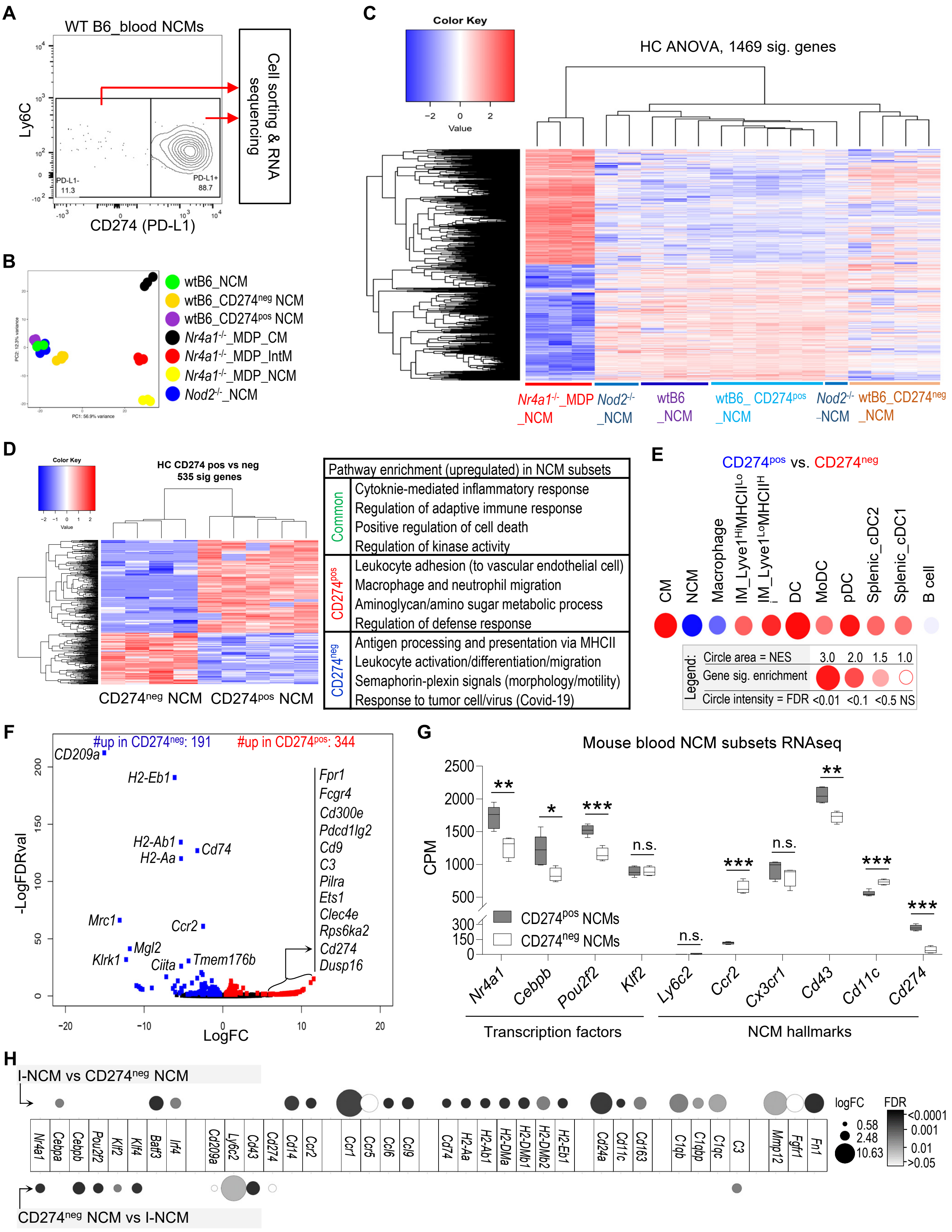

Fig.S8. *Transcriptional profiling of steady-state PD-L1^{pos} and PD-L1^{neg} NCM subsets in wtB6 mouse blood.*

A) Experimental scheme showing the steady-state PD-L1^{pos} and PD-L1^{neg} NCM subsets were sorted by flow cytometry from wtB6 mice blood for subsequent RNA extraction and bulk RNA sequencing.

(**B**) PCA plot shows separation of different monocyte subsets.

(**C**-**G**) Differential gene expression pattern between steady-state PD-L1^{pos} NCMs and PD-L1^{neg} NCMs revealed by heatmap analysis (**C**), Metascape pathway enrichment analysis (**D**) and GSEA (**E**), volcano plot (**F**) and pairwise comparison of gene expression between PD-L1^{pos}- and PD-L1^{neg}-NCM subsets (**G**). Partial of pathway enrichment pattern (**D**) and typical transcriptional markers (**F-G**) for MDP-inducible I-NCMs were detected in steady-state PD-L1^{neg} NCM subset. In (G), RNAseq CPM data are presented as the mean ± SEM; n=4-5 in each group; *p < 0.05, **p < 0.01, ***p < 0.001; 2-tailed t test.

(**H**) Weaker expression of typical I-NCM gene signatures in steady-state PD-L1^{neg} NCMs was revealed in enrichment bubble plot by pairwise comparison between I-NCMs and steady-state PD-L1^{neg} NCMs.

Supplemental Fig.9

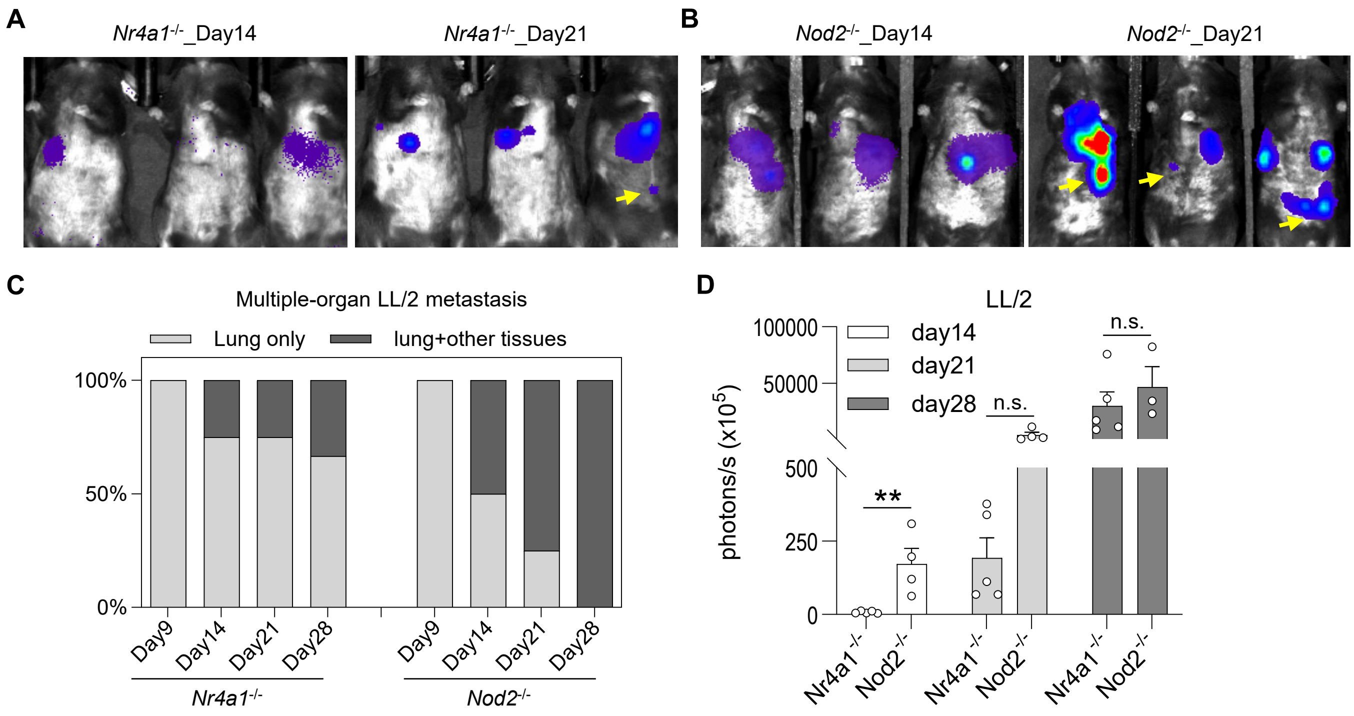

Fig.S9. *Genetic Nod2 depletion promotes LL/2 lung metastatic colonization and metastatic spread to other tissues.*

(**A-B**) Representative LAGO images showing accumulation and colonization of LL/2-LUC ($3x10^5$ cells per mouse) in lung and other tissues at indicated timepoints after tail vein injection into *Nr4a1*$^{-/-}$ mice (**A**) or *Nod2*$^{-/-}$ mice (**B**).

(**C-D**) Distribution (**C**) and quantification (**D**) of LL/2-LUC cells detected and determined by LAGO at indicated time points in *Nr4a1*$^{-/-}$ mice (**A**) or *Nod2*$^{-/-}$ mice (**B**). In (**D**), data are presented as the mean ± SEM; n=4-5 in each group; *p < 0.05, **p < 0.01; 2-tailed t test.

Supplemental Fig.10

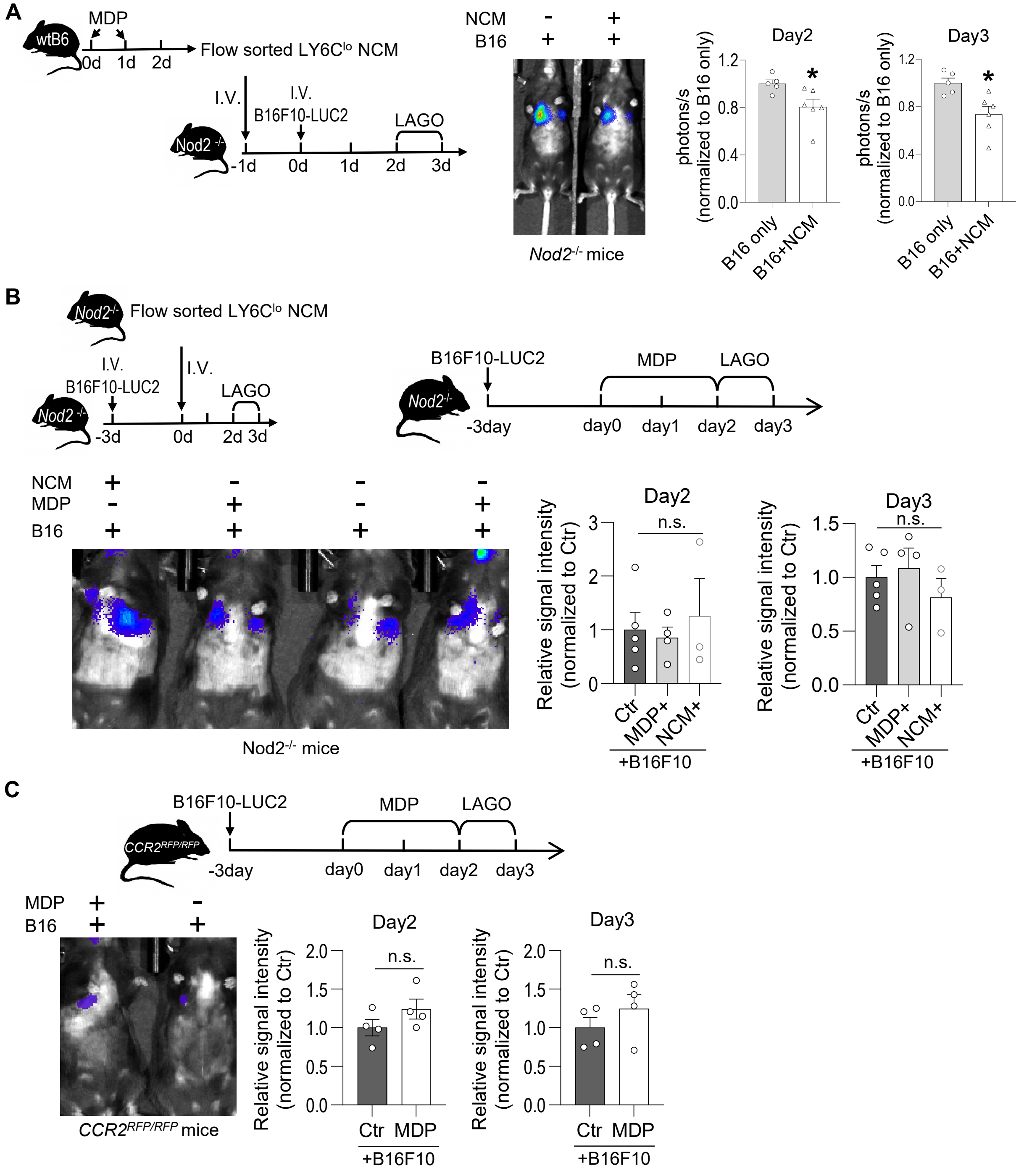

Fig.S10. Effect of reconstitution of chimeric $LY6C^{lo}$ monocyte subsets or MDP treatment in *Nod2*$^{-/-}$ or *CCR2*$^{RFP/RFP}$ mice. (**A**) Reconstitution of $1X10^5$ chimeric $LY6C^{lo}$ monocyte subsets sorted from MDP-treated wild-type B6 mice (the mixture of I-NCMs and dominant N-NCMs) in *Nod2*$^{-/-}$ mice suppresses the colonization of the established metastatic B16F10 melanoma cells in lung. (**B**) Reconstitution of $1X10^5$ $LY6C^{lo}$ monocyte subsets sorted from *Nod2*$^{-/-}$ mice or MDP-treatment in *Nod2*$^{-/-}$ mice did not suppress the colonization of the established metastatic B16F10 melanoma cells in lung. (**C**) MDP-treatment in *Ccr2*$^{RFP/RFP}$ mice did not suppress the colonization of the established metastatic B16F10 melanoma cells in lung. Data are presented as the mean ± SEM; n=3-6 in each group; *$p < 0.05$; 2-tailed t test was used for (**A**) and (**C**), 1-way ANOVA test for (**B**).

Supplemental Fig.11

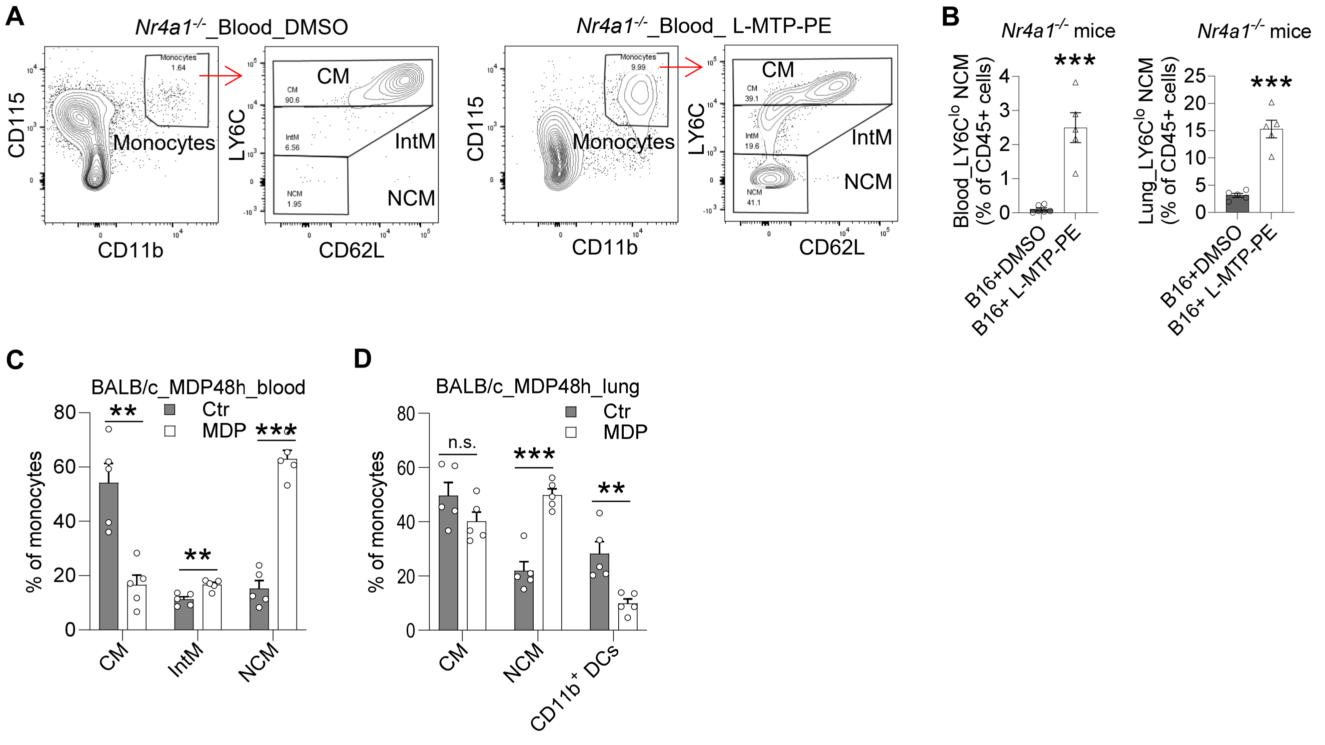

Fig.S11. *Pharmacological induction of I-NCM.*

(**A**) Representative flow cytometry plots showing gating strategy for mouse blood monocyte subsets after DMSO control (left panel) or L-MTP-PE treatment (right panel) in *Nr4a1*$^{-/-}$ mice.

(**B**) L-MTP-PE treatment increased I-NCM in the blood and lung in *Nr4a1*$^{-/-}$ mice (n=5-6).

(**C-D**) MDP-treatment induced I-NCM in BALB/c mouse blood (**C**) and lungs (**D**).

In (**B-D**), data are presented as the mean ± SEM; n=5-6 in each group; *p < 0.05, **p < 0.01, ***p < 0.001; 2-tailed t test,

Supplemental Fig.12

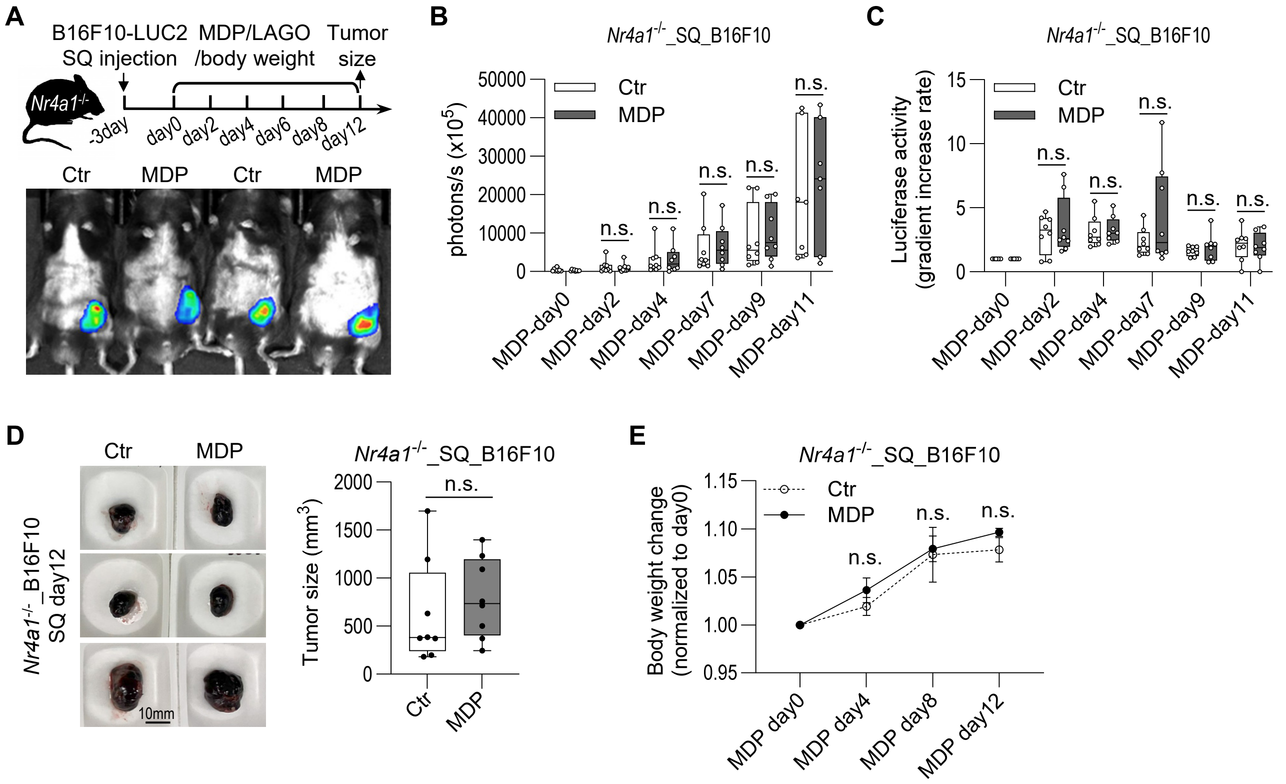

Fig.S12. _MDP does not suppress avascular subcutaneously injected melanoma colonies._

(**A**) experimental schematic and representative images of LAGO detection of luciferase activity of subcutaneous B16F10-LUC2 cells.

(**B-C**) Quantification of luciferase activity of subcutaneous B16F10-LUC2 tumor cells detected by LAGO in (**A**).

(**D**) Representative images and quantification of the tumor size of the subcutaneous B16F10-LUC2 tumors 12 days after subcutaneously injection at left flank. Scale bar=10 mm.

(**E**) Body weight change plot showing the body weight change of mice in subcutaneous B16F10 mouse model.

In (**B-E**), data are presented as the mean ± SEM; n=4-8 in each group; *p < 0.05, **p < 0.01, ***p < 0.001; 2-tailed t test,

Supplemental Fig.13

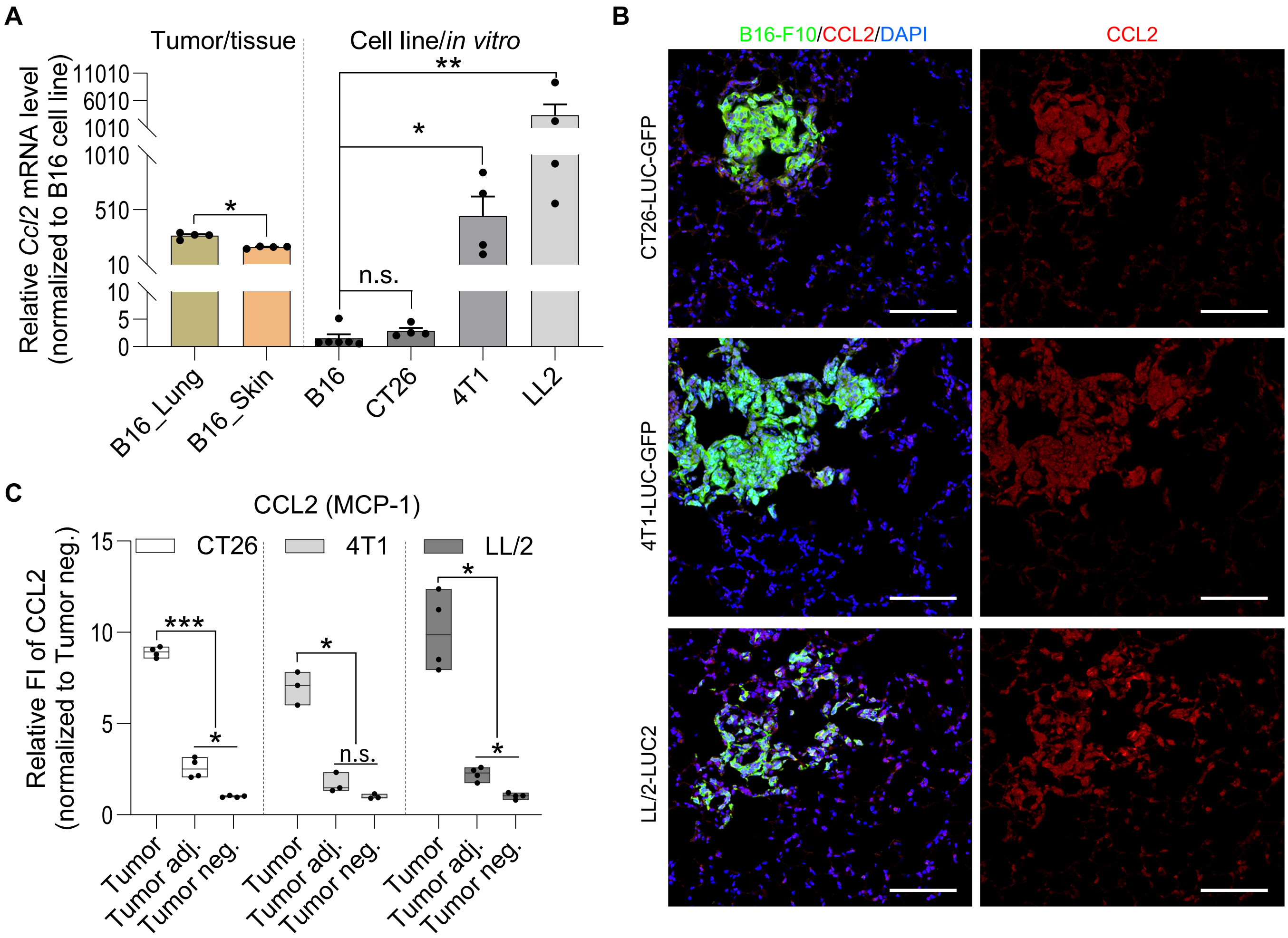

Fig.S13. *Detection of CCL2 expression in cancer cells.*

(**A**) mRNA level of *CCL2* detected by qPCR in cultured B16F10-LUC2, CT26-LUC-GFP, 4T1-LUC-GFP or LL/2-LUC2 cell lines (right part), and in metastasized or subcutaneously (SQ) implanted B16F10-LUC2 cancer cells that were isolated at 10-14 days post IV injection or SQ injection, respectively (left part). Data are presented as Mean ± SEM; n=4-6 in each group; *p < 0.05, **p < 0.01; 1-way ANOVA test (for three-column data sets) and 2-tailed t test (for two-column data sets).

(**B**) Protein level of CCL2 detected by IHC in mouse lung at 10-14 days post IV injection of CT26-LUC-GFP or 4T1-LUC-GFP in BALB/c mice, or LL/2-LUC2 cells in *Nr4a1*$^{-/-}$ mice. B16F10 cell clusters were detected by anti-GFP antibody for CT26-LUC-GFP and 4T1-LUC-GFP, or by anti-luciferase for LL/2-LUC2. Scale bar=100 μm.

(**C**) The relative fluorescence intensity (FI) of CCL2 in 8-12 areas from multiple slides from 3-4 mice in each group was measured by imageJ and quantified. Data are presented as Mean ± SEM; n=3-4 in each group; *p < 0.05, **p < 0.01, ***p < 0.001; 1-way ANOVA test.

Supplemental Fig.14

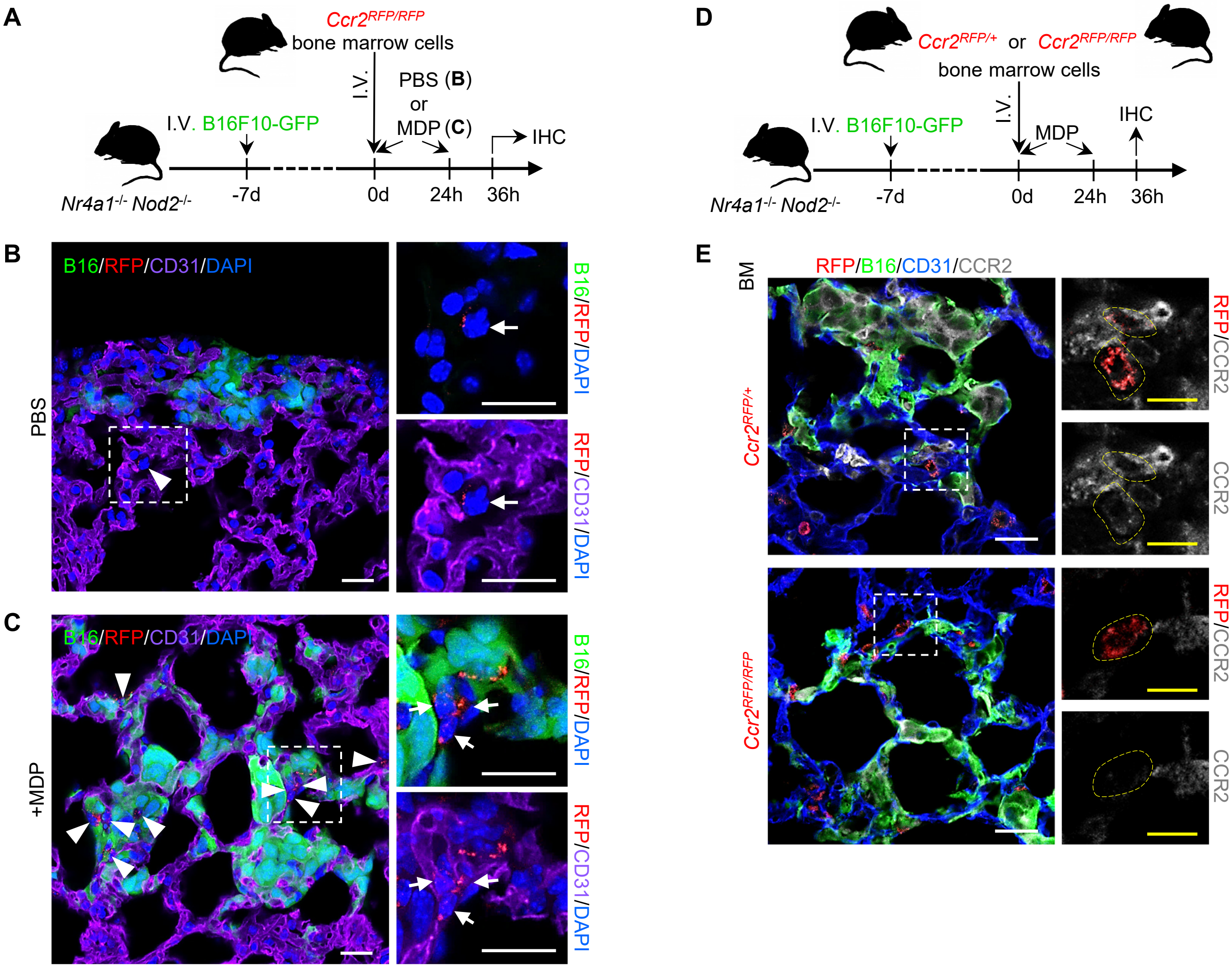

Fig.S14. *Detection and colocalization of RFP^+ cells from adoptively transferred $Ccr2^{RFP/RFP}$ or $Ccr2^{RFP/+}$ mouse BM cells with the established B16F10-GFP clusters in lung in $Nr4a1^{-/-}$ $Nod2^{-/-}$ mice.*

(**A-C**) Experimental design diagram (**A**) and representative confocal images (**B-C**) showing negative detection of RFP^+ cells around the established B16F10-GFP cluster in lung sections of *$Nr4a1^{-/-}$ $Nod2^{-/-}$* mice after adoptive transfer of BM cells obtained from *$Ccr2^{RFP/RFP}$* mice (**B**), with RFP^+ cells accumulating in blood vessel (CD31+) after MDP treatment (**C**). Scale bar= 20 μm.

(**D-E**) As described in experimental design diagram in (**D**), the BM cells prepared from *$Ccr2^{RFP/RFP}$* or *$Ccr2^{RFP/+}$* mice were retro-orbitally injected into *$Nr4a1^{-/-}$ $Nod2^{-/-}$* mice on day 7 following retro-orbital injection of B16F10-GFP cells. The MDP or PBS control treatment started immediately after injection of BM and continued for 2 days prior to IHC detection. In (**E**), representative confocal images show that the expression of CCR2 can be detected in RFP^+ monocytes from adoptively transferred *$Ccr2^{RFP/+}$* mouse BM cells (**E**, upper panel, circled), but not from *$Ccr2^{RFP/RFP}$* mouse BM cells (**E**, lower panel, circled), which were located around or within the established extravasated or intravascular B16F10-GFP clusters in *$Nr4a1^{-/-}Nod2^{-/-}$* mouse lung after MDP treatment. White and yellow scale bar= 20 and 10 μm, respectively,

Supplemental Fig.15

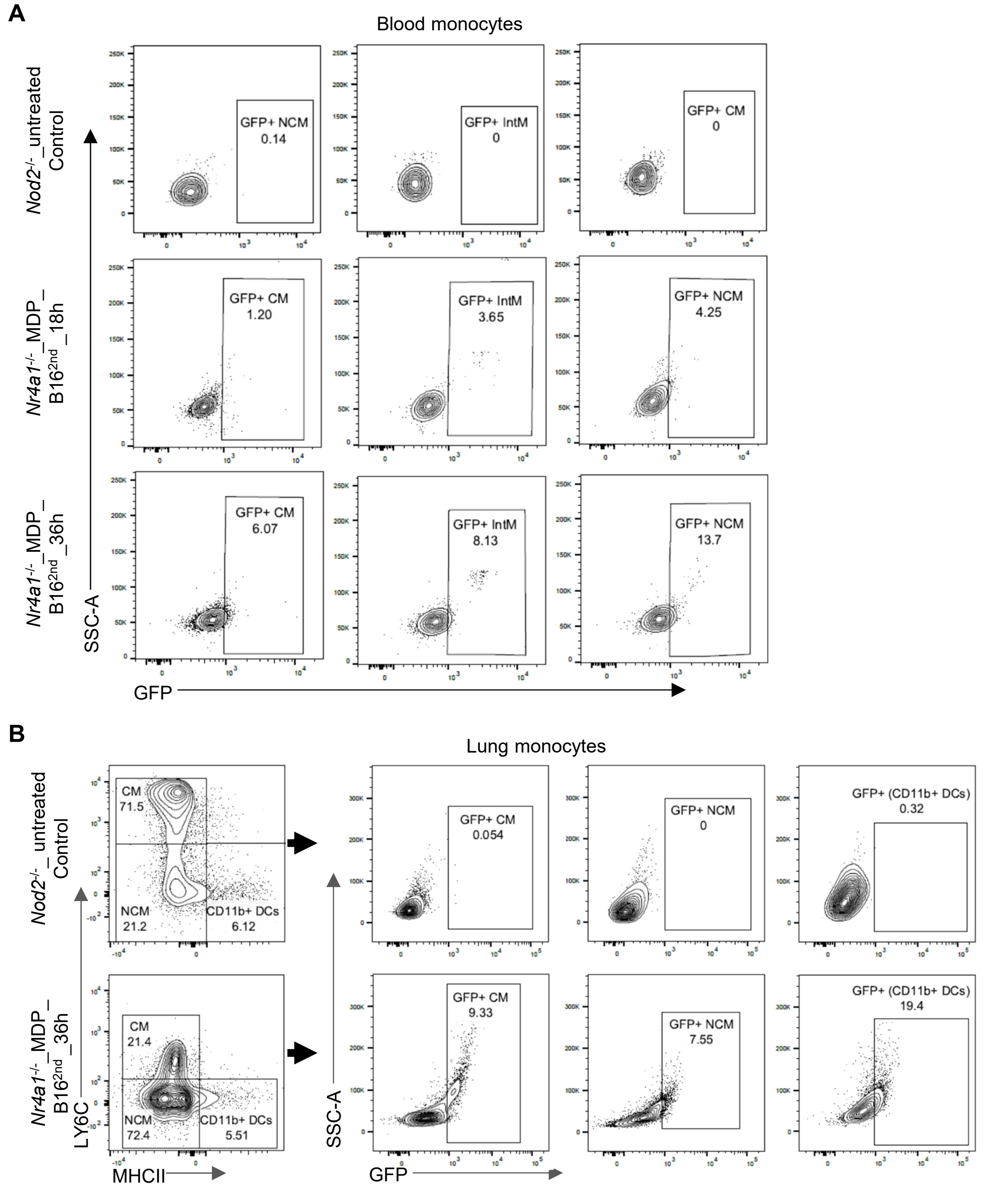

Fig.S15. *Detection of in vivo B10F10 material uptake by I-NCM by flow cytometry.*

(**A-B**) Representative gating and detection of GFP positive LY6C^{lo} monocytes in blood (**A**) or lung (**B**) in *Nr4a1$^{-/-}$* mice after retro-orbital injection of MDP and B16F10-GFP at indicated time points.

Supplemental Fig.16

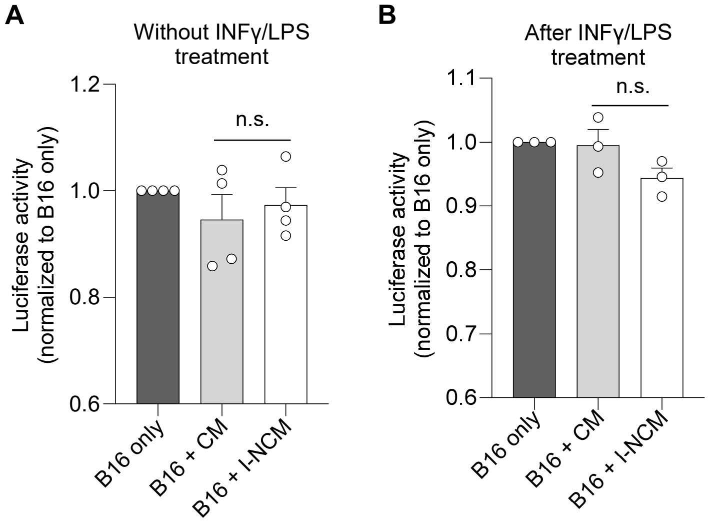

Fig.S16. *Detection of direct tumor killing effect of NOD2-dependent LY6C^{lo} NCMs.*

(**A-B**) Direct B16F10-LUC2 killing effects of monocyte subsets by flow cytometry. The cells were sorted from MDP-treatment *Nr4a1*$^{-/-}$ mice and stimulated without (**A**) or with (**B**) LPS/IFNγ for 24h to promote activation, followed by co-culture with B16F10-LUC2 cells at 50:1 monocyte to tumor cell ratio (25000: 500) in 96-well plate for 24h.

Data are presented as Mean ± SEM; n=3-4 in each group; *p < 0.05; 2-tailed t test.

Supplemental Fig.17

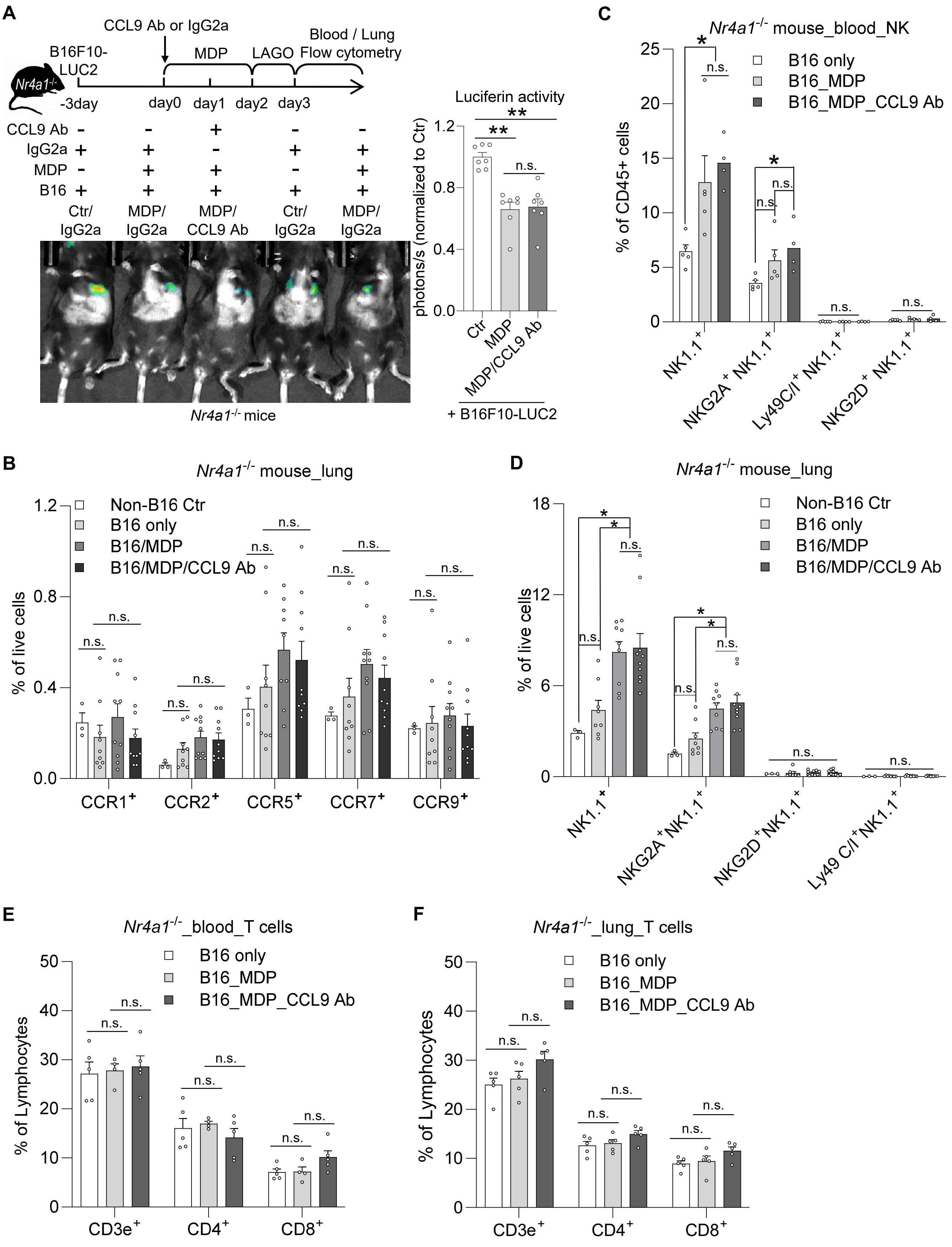

Fig.S17. *CCL9 does not contribute to I-NCM dependent and NK-mediated tumor regression*.

(**A**) Neutralization of CCL9 by anti-CCL9 antibody did not affect the MDP-mediated attenuation of B16F10 colonization in *Nr4a1*$^{-/-}$ mice.

(**B**) Detection of chemokine receptors on NK cells by flow cytometry in *Nr4a1*$^{-/-}$ mice lung.

(**C-D**) Detection of NK cell activating/inhibitory/licensing receptors by flow cytometry in *Nr4a1*$^{-/-}$ mice blood (**C**) and lung (**D**).

(**E-F**) MDP-treatment or anti-CCL9 antibody could not affect T cells in *Nr4a1*$^{-/-}$ mice blood (**E**) and lung (**F**).

In (**B-D**), the NK cells were gated as CD3e^{-} Ly6G^{-} LY6C^{-} CD45^{+} NK1.1^{+} in flow cytometry analysis.

Data are presented as Mean ± SEM; n=3-10 mice in each group; *p < 0.05; **p < 0.01, ***p < 0.001; Kruskal-Wallis test was used for **A** and **D**: NK1.1; 1-way ANOVA test for the rest comparison.

Supplemental Fig.18

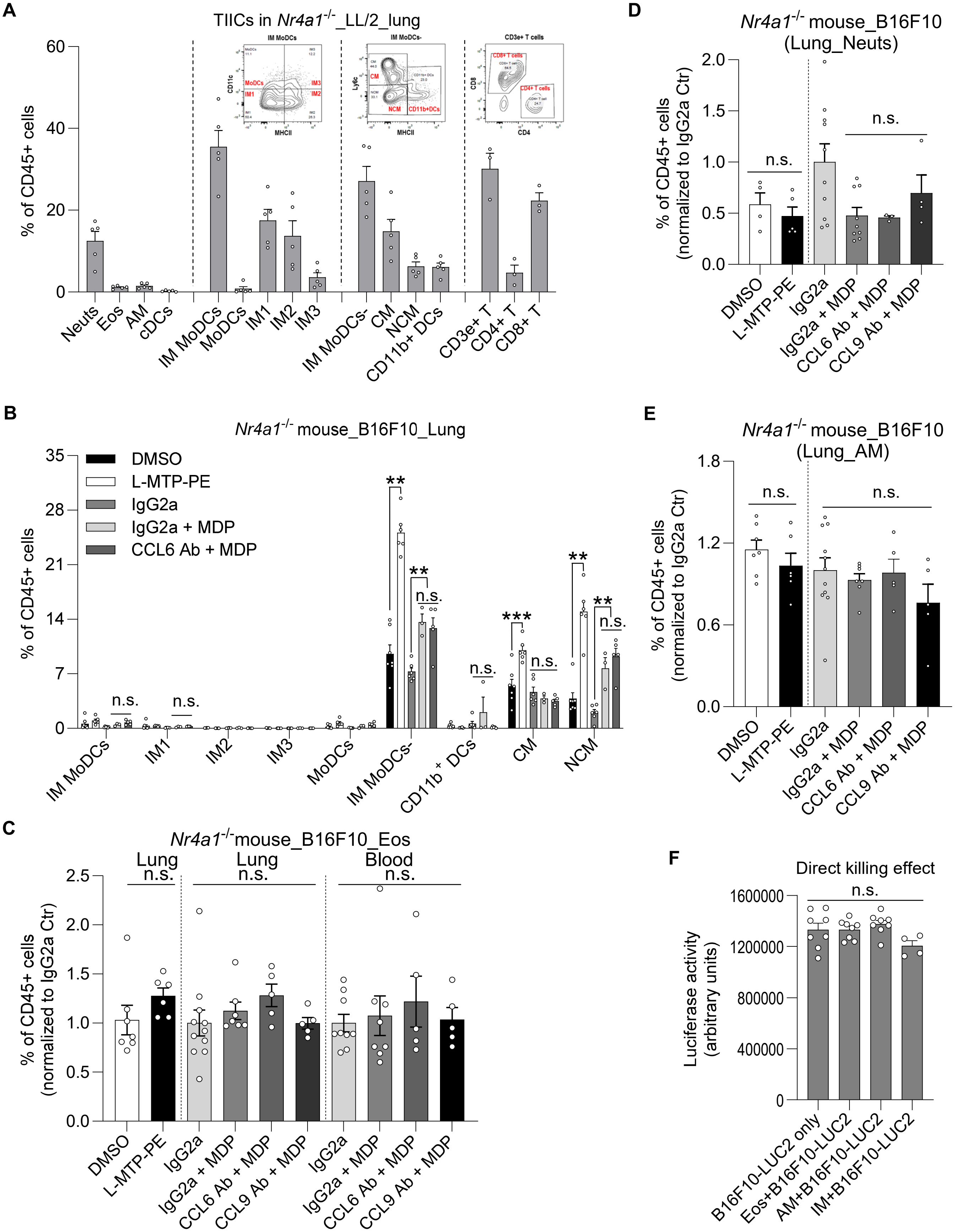

Fig.S18. *CCL6 and CCL9 do not alter immune cells in tumor microenvironment*.

(**A**) Detection of tumor infiltrating immune cells (TIICs) by flow cytometry in lung metastasized LL/2 cell clusters in *Nr4a1*$^{-/-}$ mice. TIICs were isolated from 3-5 mice at 4 weeks after tail vein injection of LL/2 cells.

(**B-E**) Detection of Monocytes, interstitial macrophages (IM), monocyte-derived dendritic cells (MoDCs) (**B**), eosinophils (Eos) (**C**), neutrophils (Neuts) (**D**) and alveolar macrophages (AM) (**E**) in *Nr4a1*$^{-/-}$ mice bearing B16F10 lung metastasis with or without MDP-treatment and/or CCL6 or CCL9 neutralization.

(**F**) Detection of direct tumor killing effect of Eos, AM and IM on B16F10-LUC2 cells. The eosinophils (Eos), alveolar macrophages (AM), and interstitial macrophages (IM), were sorted from lungs from five wtB6 mice, followed by co-culture with B16F10-LUC2 cells at 6:1 immune cells to tumor cell ratio (9000: 1500) in 96-well plate for 24h.

Data are presented as Mean ± SEM; n=3-10 in each group; *p < 0.05, **p < 0.01; 1-way ANOVA test (for 3-column datasets) and 2-tailed t test (for 2-column datasets).

Supplemental Fig.19

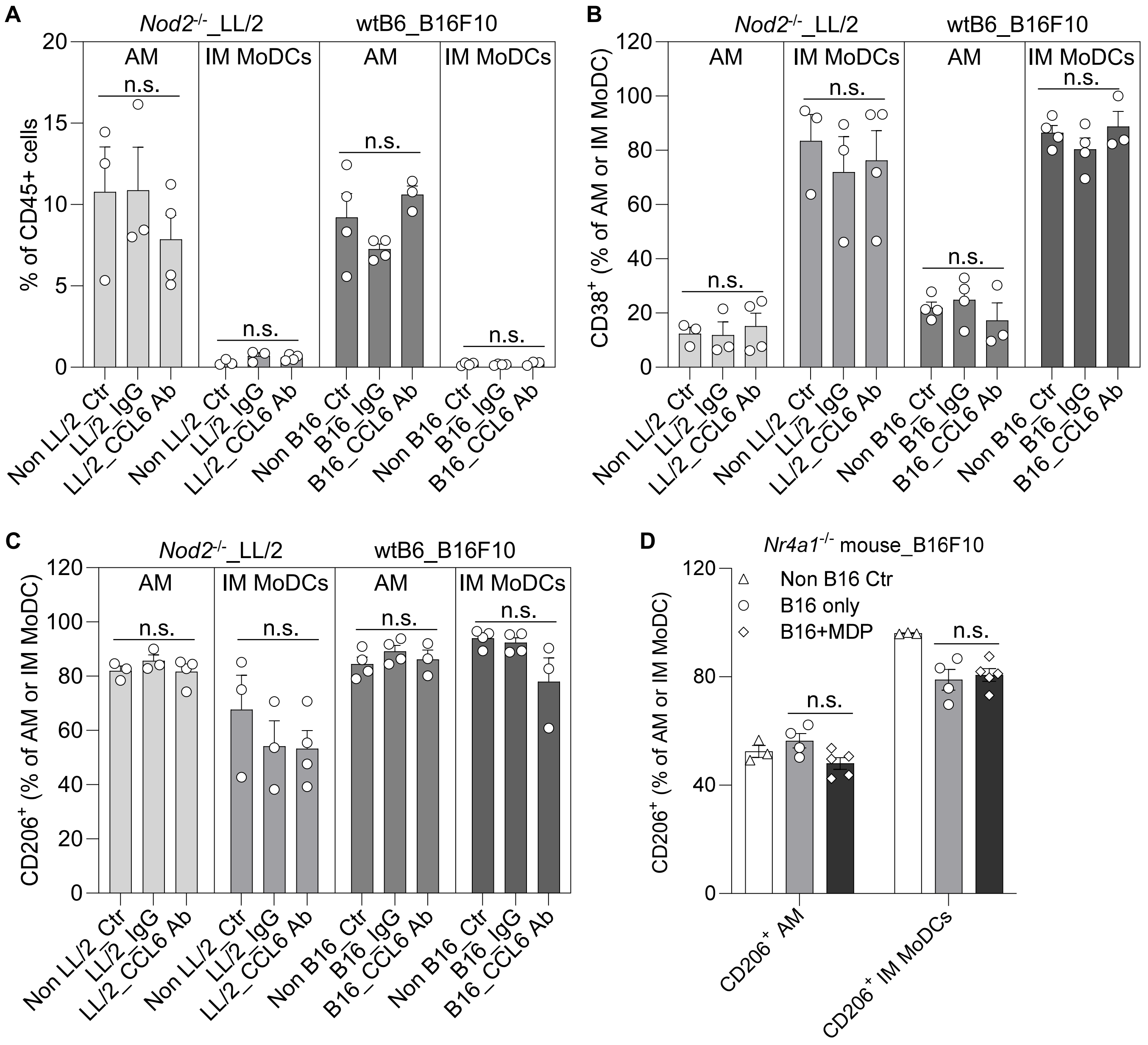

Fig.S19. *CCL6 neutralization or MDP-treatment does not alter macrophage polarization*.

(**A**-**C**) Detection of the polarization of lung alveolar macrophages (AM) and interstitial macrophages (IM) in *Nod2*$^{-/-}$ and wtB6 mice bearing LL/2 and B16F10 lung metastasis, respectively. The *Nod2*$^{-/-}$ and wtB6 mice were intravenously injected with 3x10^5 LL/2 and B16F10 cells, respectively. Anti-CCL9 antibody was injected intravenously on day 3 after the injection of cancer cells. The lung samples were collected for flow cytometry analysis of macrophage polarization markers at 36h post the injection of anti-CCL9 antibody.

(**D**) Detection of CD206$^+$ AM and IM in *Nr4a1*$^{-/-}$ mice bearing B16F10 lung metastasis. The *Nr4a1*$^{-/-}$ mice were intravenously injected with 3x10^5 B16F10 cells. MDP was injected intravenously on day3 after the injection of cancer cells. The lung samples were collected for flow cytometry analysis of the expression of CD206 at 36h post the injection of MDP.

Data are presented as Mean ± SEM; n=3-5 in each group; *p < 0.05; 1-way ANOVA test.

Supplemental Fig.20

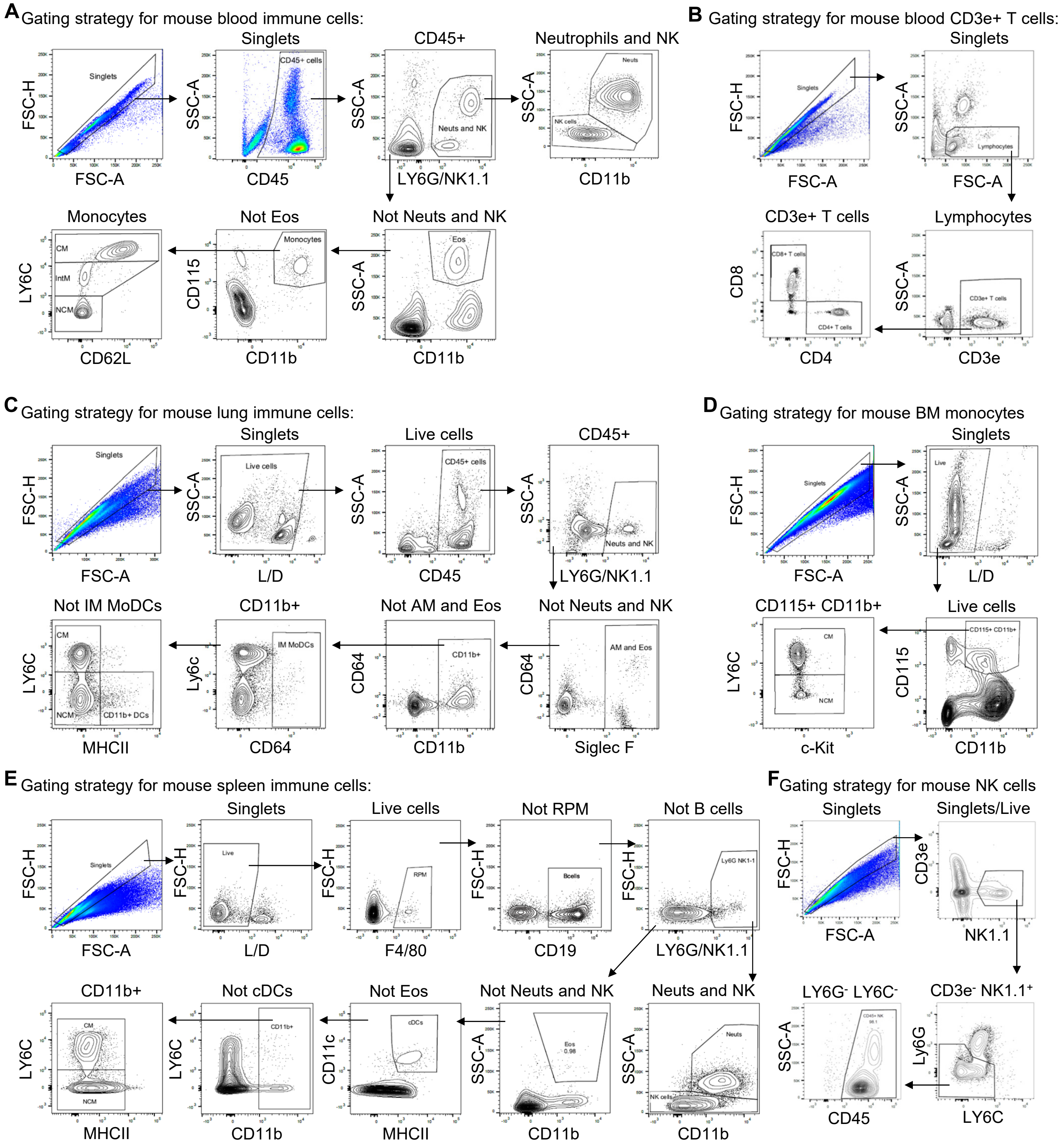

Fig.S20. *Gating strategy for flow cytometry analysis of mouse monocytes and other immune cells.*

(**A-F**) The layout of gating strategy was edited and made using FlowJo (v10.10).

Movie S1: Movie from two-photon imaging showing the accumulation of Hoechst33342-stained NOD2-dependent LY6C^{lo} monocytes (blue) around or within the established B16F10-GFP metastatic clusters (green) in the lung within 1h after IV monocytes injection. Vasculature was labeled with Dextran-Rhodamine (Red).

Table S1: List of selected 20 marker genes (FDR<0.05) that are upregulated in NOD2-dependent LY6C^{lo} I-NCM and commonly expressed in both mouse and human blood nonclassical monocytes.

Table S2: List of 2408 genes ((>1.5-fold) that are all upregulated in mouse blood NOD2-dependent LY6C^{lo} I-NCM (>1.5-fold) compared to NR4A1-dependent LY6C^{lo} N-NCM.

Table S3: Antibodies used for flow cytometry

Table S4: Primers used for qPCR.
